# The Novel Viral Non‐Coding RNA LMP2A‐IT1 Promotes the Lytic Replication of EBV

**DOI:** 10.1002/advs.78488

**Published:** 2026-11-05

**Authors:** Zheng‐Zhou Lu, Jun‐Fei Wu, Nannan Zhu, Ming‐Hui Yang, Le‐Le Zhang, Yuan‐Tao Liu, Wei‐Jun Chen, Jin‐Kun Tang, Jia‐Wei Ge, Chao‐Bo Lei, Tao Zhang, Yu Wang, Chao‐Yun Huang, Qian Zhong, Mu‐Sheng Zeng, Yan Li

**Affiliations:** ^1^ State Key Laboratory of Oncology in South China Guangdong Key Laboratory of Nasopharyngeal Carcinoma Diagnosis and Therapy Sun Yat‐sen University Cancer Center Guangzhou China; ^2^ State Key Laboratory of New Targets Discovery and Drug Development for Major Diseases Gannan Innovation and Translational Medicine Research Institute Gannan Medical University Ganzhou China; ^3^ Department of Clinical Laboratory State Key Laboratory of Oncology in South China Guangdong Provincial Clinical Research Center for Cancer Sun Yat‐sen University Cancer Center Guangzhou China; ^4^ Department of Pathology Sun Yat‐sen University Cancer Center Guangzhou China; ^5^ Guangdong‐Hong Kong Joint Laboratory for RNA Medicine Sun Yat‐sen University Guangzhou China

**Keywords:** EBV, EBV‐encoded RNA, glycoRNA, LMP2A‐IT1, lytic replication

## Abstract

The Epstein‐Barr virus (EBV) is recognized as the first human tumor virus and etiologically linked to a spectrum of malignancies and autoimmune diseases. EBV lytic replication contributes substantially to pathogenesis, but the viral components that enable efficient replication within host cells remain incompletely understood. Here, we identified a previously unknown EBV non‐coding RNA (ncRNA) from glycoRNA samples and designated it LMP2A‐IT1. This viral ncRNA was consistently detected across multiple EBV‐infected cell lines, in exosomes, and in EBV‐positive nasopharyngeal carcinoma tissues, exhibiting a predominantly cytoplasmic distribution. Functional analyses reveal that the induction of LMP2A‐IT1 during EBV lytic replication actively drives the lytic cycle, resulting in a self‐amplifying positive feedback loop. Mechanistically, LMP2A‐IT1 appears to activate an inflammatory response, which subsequently upregulates BZLF1, a master regulator of the EBV lytic cascade, to facilitate lytic progression. Together, our findings suggest that LMP2A‐IT1 represents a previously unrecognized viral factor that EBV deploys to achieve robust replication. To our best knowledge, this is the first report of a viral RNA harboring glycosylation modifications, thereby expanding the glycoRNA landscape. Moreover, this study offers new mechanistic insights into EBV replication as well as a potential biomarker and therapeutic target for EBV‐associated diseases.

## Graphical abstract

Decades after its discovery, EBV's genome still harbours unannotated “dark matter”. LMP2A‐IT1, a novel EBV‐encoded transcript identified in this work, is strongly upregulated during lytic replication and promotes BZLF1 expression, likely through activation of inflammatory responses, thereby enhancing viral propagation in a self‐reinforcing positive feedback loop.

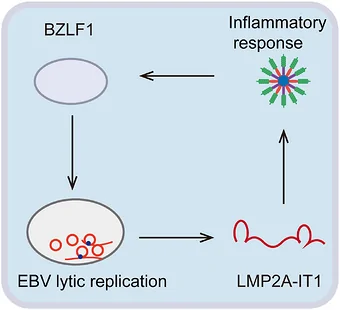

## 1 Introduction

The Epstein‐Barr virus (EBV) is a human gamma‐herpesvirus with double‐stranded DNA genome of approximately 170 kb in length, which encodes more than 80 genes [1, 2]. It is well‐known as the first human oncogenic virus to be discovered and is etiologically linked to diverse malignancies, including lymphomas, nasopharyngeal carcinoma, as well as gastric cancer [3, 4].

As a persistent virus, EBV exhibits two distinct life cycles phases, encompassing latent infection and lytic replication [5]. During latency, the virus persists in host cells by expressing a limited set of latency‐associated gene products to maintain viral genome stability during cell division. In the presence of specific stimuli, EBV transitions into lytic replication, activating the full viral transcriptional program to produce infectious virions that disseminate systemically [5, 6]. While EBV latency is well‐established as a driver of cellular transformation and tumorigenesis, there is also accumulating evidence for a critical role of lytic replication in tumor progression [7, 8]. Additionally, EBV lytic reactivation is associated with the transition from preclinical to clinical systemic lupus erythematosus (SLE) and the level of disease activity [9, 10]. Thus, EBV lytic replication is not only a mechanism for viral dissemination but also a pivotal contributor to pathogenesis. Accordingly, targeting lytic replication represents a promising therapeutic strategy for EBV‐associated cancers [11, 12].

EBV lytic replication is an ordered process mediated by the sequential expression of viral genes. It is initially dependent on the viral lytic switch proteins Zta and Rta, which are encoded by the immediate‐early genes BZLF1 and BRLF1, respectively [13]. As a viral transcription factor, BZLF1 generally forms homodimers that directly bind to BZLF1‐responsive elements in the promoters of early lytic genes to induce their expression [14, 15]. In addition, BZLF1 also binds the origin of lytic replication (ori‐Lyt) and recruits the DNA synthesis machinery to initiate viral genome replication [16, 17]. Concurrently, BRLF1, a transactivator with homology to cellular helicases, directly activates early lytic gene promoters by binding BRLF1‐responsive elements and recruits host transcriptional coactivators to drive the expression of early lytic genes [6]. The combined action of BZLF1 and BRLF1 activates other viral lytic genes eventually inducing the lytic replication cycle [13, 18, 19]. However, lytic replication makes the virus more vulnerable to immune‐mediated destruction, and EBV utilizes a network of viral and host‐derived factors to impose tight regulatory constraints on its replication machinery. Although multiple host or viral regulators have been discovered (as summarized in review [18]), the regulatory mechanisms governing EBV lytic replication remain incompletely understood. Accordingly, the potential discovery of new viral factors regulating EBV replication warrants further studies.

Glycosylated RNAs (glycoRNAs) are a newly discovered class of biomolecules that are widely distributed in different cell types, where they can be detected on the cell surface as well as in other cellular compartments [20, 21]. We recently confirmed the presence of glycoRNAs in EBV‐infected cells and established the Clier‐seq method, which uses click chemistry‐based enrichment of glycoRNAs followed by sequencing to identify novel cellular glycoRNAs [22].

In the present study, we turned our attention to the EBV genome. Notably, a previously uncharacterized transcript originating from EBV was also found to carry glycosylation modifications. This viral glycoRNA was detected in multiple EBV‑positive cell lines, in exosomes, and in EBV‐positive nasopharyngeal carcinoma patient‐derived xenograft (PDX) tissues. Functional studies revealed that this uncharacterized viral transcript acts as a regulator that promotes EBV lytic replication. Together, these findings identify a novel transcript from the EBV genomes and provide evidence that viral RNAs can be glycosylated.

## 2 Results

### 2.1 Identification of a Novel EBV‐Encoded glycoRNA Transcript

We recently demonstrated the presence of glycoRNAs in human epithelial and lymphocyte cells. To further investigate whether glycoRNAs are also present in cell‐derived exosomes and whether EBV infection influences RNA glycosylation, we collected total RNA from both EBV‐negative and ‐positive cell lines, as well as from corresponding exosome fractions, and applied the previously established metabolic labeling method to detect glycoRNAs [21, 22]. Specifically, a panel of EBV‐negative and EBV‐positive cell lines was treated with Ac4ManNAz, a sialic acid precursor bearing an azide group. After 48 h, both the cells and their secreted exosomes were harvested. Total RNA was extracted from each sample and subjected to a click chemistry reaction, which covalently conjugated biotin to the azide‐labeled glycoRNAs. The biotinylated RNAs were then detected by RNA blotting using streptavidin‐horseradish peroxidase (HRP).

Our results showed that both cellular and exosomal RNAs were readily recognized by streptavidin‐HRP, producing clear blotting signals, indicating that both pools contain glycoRNAs. Notably, despite equal RNA loading (as confirmed by ethidium bromide staining), exosomal RNA derived from EBV‐positive cells consistently exhibited much stronger blotting signals than that from EBV‐negative cells. This trend was observed across multiple cell lines, including CNE2, HK‐1, HNE1, and P3HR1(Figure 1A–D). These observations raised two possibilities either that EBV promotes the secretion of host‐encoded glycoRNAs into exosomes, or that EBV itself may contribute its own glycoRNAs to the exosomal fraction.

**FIGURE 1:**
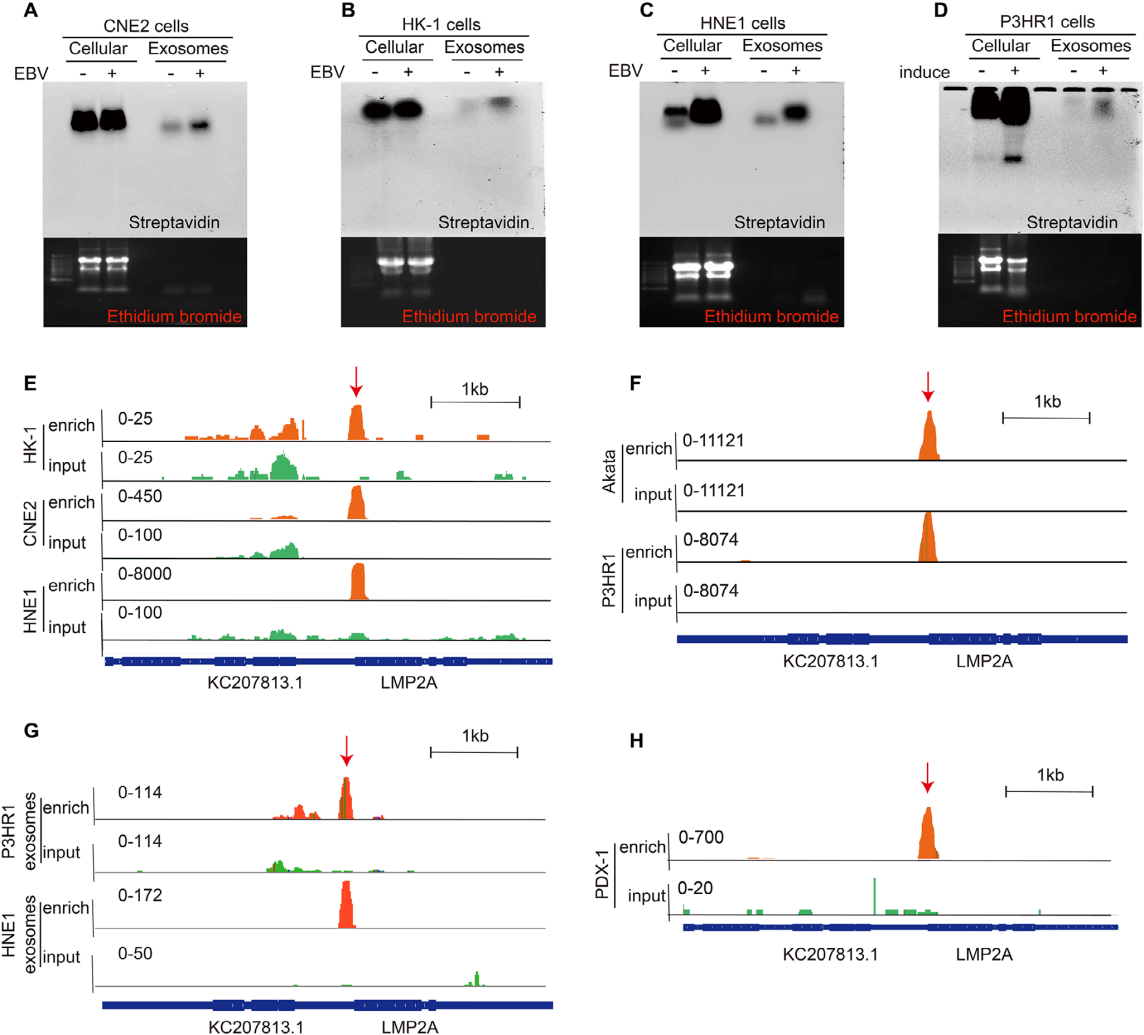
Identification of a novel EBV‐encoded RNA in glycoRNA samples. (A‐D) RNA blotting of glycoRNAs in total RNA from cell lines and exosomes. Cellular or exosomal RNA from CNE2 cells (A), HK‐1 cells (B), HNE1 cells (C) or P3HR1 cells (D) was biotin‐labeled, then subjected to agarose gel electrophoresis, transferred to a nitrocellulose membrane, and subsequently detected using streptavidin‐HRP. The lower pictures show the bands visualized through ethidium bromide staining before the membrane transfer. (E‐H) Integrative Genomics Viewer (IGV) diagram showing a remarkable peak in the unannotated region of the EBV genome. RNA from the indicated cell lines, exosomes or PDX was sequenced, and the data were processed and visualized using IGV. The horizontal axis represents the location on the EBV genome, and the vertical axis represents the read count. Numbers in the track, such as 0–25, indicate the values on the vertical axis. “input” represents total RNA, and “enrich” represents glycoRNA enriched from the total RNA.

To determine whether these glycoRNAs include EBV‐encoded species, we enriched glycoRNAs from cells, exosomes, and PDX tissues using streptavidin‐coated beads, followed by sequencing and analysis using our previously established Clier‐seq pipeline [22]. Notably, a portion of the reads could be unambiguously aligned to the EBV genome (reference strain KC207813.1), indicating that viral RNAs also undergo glycosylation. Among these viral glycoRNAs, a particularly abundant transcript was mapped to position 167,123 bp of the EBV genome. This glycoRNA was consistently detected across multiple EBV‐positive epithelial cell lines (CNE2, HK1, HNE1), EBV‐positive lymphocyte lines (Akata, P3HR1), exosomes derived from EBV‐positive cells (HNE1, HR1), as well as PDX tissues (Figure 1E–H). The corresponding sequence had not been previously annotated or reported, suggesting that it represents a newly discovered EBV RNA. We therefore decided to focus our subsequent studies on this transcript.

### 2.2 LMP2A‐IT1 is a 276nt RNA That is Conserved Among EBV Strains

Using 5’ and 3’ rapid amplification of cDNA ends (RACE), we confirmed the actual existence of this bona fide transcript (Figure 2A) and determined that it has an exact length of 276 nt (Figure S1). Based on annotation in the EBV genome, this transcript encoded within the first intron of the LMP2A gene (Figure 2B), and thus it was named LMP2A‐IT1 according to the Guidelines for Naming Human Non‐Coding RNAs [23]. The secondary structure predicted by RNAfold [24] showed multiple stem‐loop structures (Figure 2B), suggesting that LMP2A‐IT1 has a stable structure that can support defined biological functions. Tertiary structure modeling using 3dRNA [25] revealed a Y‐shaped fold with three‐way junction architecture formed by double helices converging at a central hub (Figure S2). This intricate architecture may also help explain the stability of LMP2A‐IT1. Notably, LMP2A‐IT1 exhibited high sequence conservation across EBV strains, except for a 30 bp insertion in the B95‐8 strain (Figure 2C), a region previously linked to oncogenicity of the LMP1 gene [26]. The evolutionary conservation of this intronic RNA underscores its potential functional significance.

**FIGURE 2:**
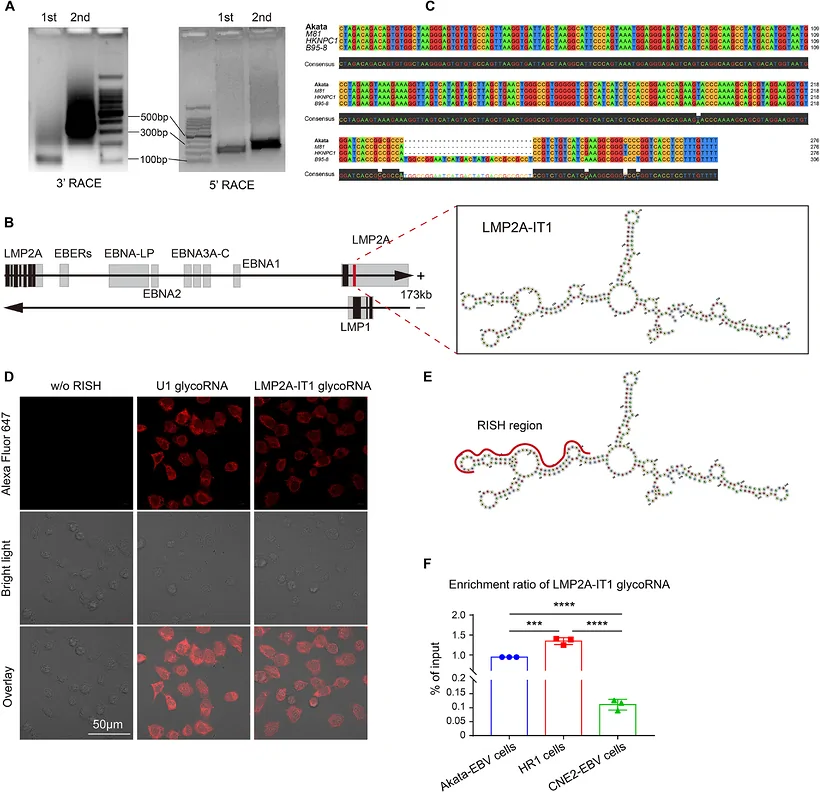
Sequence‐ and glycosylation analysis of LMP2A‐IT1. (A) Agarose gel electrophoresis results of RACE products. Fresh RNA extracted from EBV‐positive CNE2 cells (CNE2‐EBV) was immediately used for 5’RACE or 3’RACE assays, after which the products of two rounds of amplification (1^st^ and 2^nd^ in A, respectively) were separated on agarose gels. (B) Simplified map of the EBV genome. The red vertical bars represent the location of LMP2A‐IT1 in the EBV genome, and the RNA secondary structure of LMP2A‐IT1 predicted by RNAfold is shown in the expanded box. (C) Conservation analysis of LMP2A‐IT1 sequences among different EBV strains. (D) Identification of LMP2A‐IT1 glycosylation by ARPLA. CNE2‐EBV cells were fixed and then subjected to dual recognition using glycan probes and RNA in situ hybridization (RISH) probes, followed by rolling‐circle amplification and detection of the amplified products with Alexa Fluor 647‐labelled fluorescent probes. Note: w/o RISH denotes the absence of the RNA in situ hybridization probe. (E) Schematic diagram of the RISH probe binding sites on LMP2A‐IT1 as depicted in panel D. (F) Enrichment ratio of LMP2A glycoRNA determined by click chemistry‐based RNA enrichment followed by qPCR (Clier‐qPCR). Akata‐EBV, HR1, and CNE2‐EBV cells were treated with Ac4ManNAz (100 µM) for 36 h. Total RNA was extracted, and glycoRNAs were biotinylated via click chemistry and enriched using streptavidin‐conjugated magnetic beads. The enrichment ratio of LMP2A‐IT1 was calculated by RT‐qPCR as the abundance in the enriched fraction relative to that in the input fraction. Data are mean ± SD. n = 3. One‐way analysis of variance (ANOVA) was carried out with Tukey's correction for multiple comparisons. ns, no significance; ***p < 0.001; ^****^ *p* < 0.0001.

Given that LMP2A‐IT1 was identified in a glycoRNA‐enriched fraction, we next investigated whether this transcript is subjected to glycosylation. To this end, we performed the sialic acid aptamer and RNA in situ hybridization‐mediated proximity ligation assay (ARPLA), a method that enables dual recognition of glycoRNAs using glycan‐ and RNA probes, thereby allowing specific detection of glycosylated individual RNA species [22, 27, 28]. Analogous to the positive control U1 glycoRNA, LMP2A‐IT1 was also readily detectable in the ARPLA (Figure 2D,E), confirming that it can be glycosylated. We further employed our previously established Clier‐qPCR method [22] to quantify the proportion of glycosylated LMP2A‐IT1 relative to total LMP2A‐IT1. The data revealed that the enrichment ratio varied among the three EBV‐positive cell lines examined and was lowest in CNE2‐EBV cells, at approximately 0.1% of input (Figure 2F). Considering this low enrichment efficiency and the technical limitations for dissecting functions of individual glycoRNA species, our subsequent experiments characterized LMP2A‐IT1 in bulk, without separating its glycosylated and non‐glycosylated isoforms. Therefore, our results characterize the role of LMP2A‐IT1 overall but cannot establish whether glycosylation contributes to the observed phenotype.

### 2.3 LMP2A‐IT1 Exhibits a Cytoplasmic Localization

As the subcellular localization of ncRNAs has significant implications for their biological functions [29], we investigated whether LMP2A‐IT1 is localized to the cytoplasm or the nucleus. Subcellular localization prediction using the LncLocator algorithm [30] indicated that LMP2A‐IT1 is predominantly cytoplasmic (Figure 3A). To experimentally validate this prediction, we performed nuclear‐cytoplasmic fractionation and extracted RNA from each fraction. Conventional RT‑PCR using random hexamer primers detected the small nuclear RNA U1 exclusively in the nuclear fraction, whereas the mitochondrially encoded mRNA of cytochrome c oxidase I (MT‐CO1) was present only in the cytoplasmic fraction (Figure 3B,D), confirming the stringency of our fractionation procedure. We then examined the distribution of LMP2A‐IT1 across the two compartments. Because the LMP2A‐IT1 transcript overlaps with the LMP2A and LMP1 genes, conventional RT‑PCR‑based detection of LMP2A‐IT1 would also amplify LMP2A pre‑mRNA and LMP1 mRNA, leading to results that do not reflect the true signal of the LMP2A‐IT1 transcript. To overcome this, we established a quantitative RT‑PCR strategy that accurately measures LMP2A‐IT1, termed gene‑specific RT‑PCR. In this procedure, reverse transcription was conducted using a LMP2A‐IT1‑specific primer (named IT1‑RT‑GSP) instead of random hexamer primers to avoid generating LMP1 cDNA. In the subsequent PCR amplification, we used primers targeting the LMP2A‐IT1 region (named LMP2A‐IT1 primers) and primers targeting a specific region of LMP2A pre‑mRNA (named LMP2A‑intron primers) on the same cDNA sample. Finally, the amplification signal obtained with the LMP2A‑intron primers was subtracted from that obtained using the LMP2A‐IT1 primers to obtain the true signal of LMP2A‐IT1.

**FIGURE 3:**
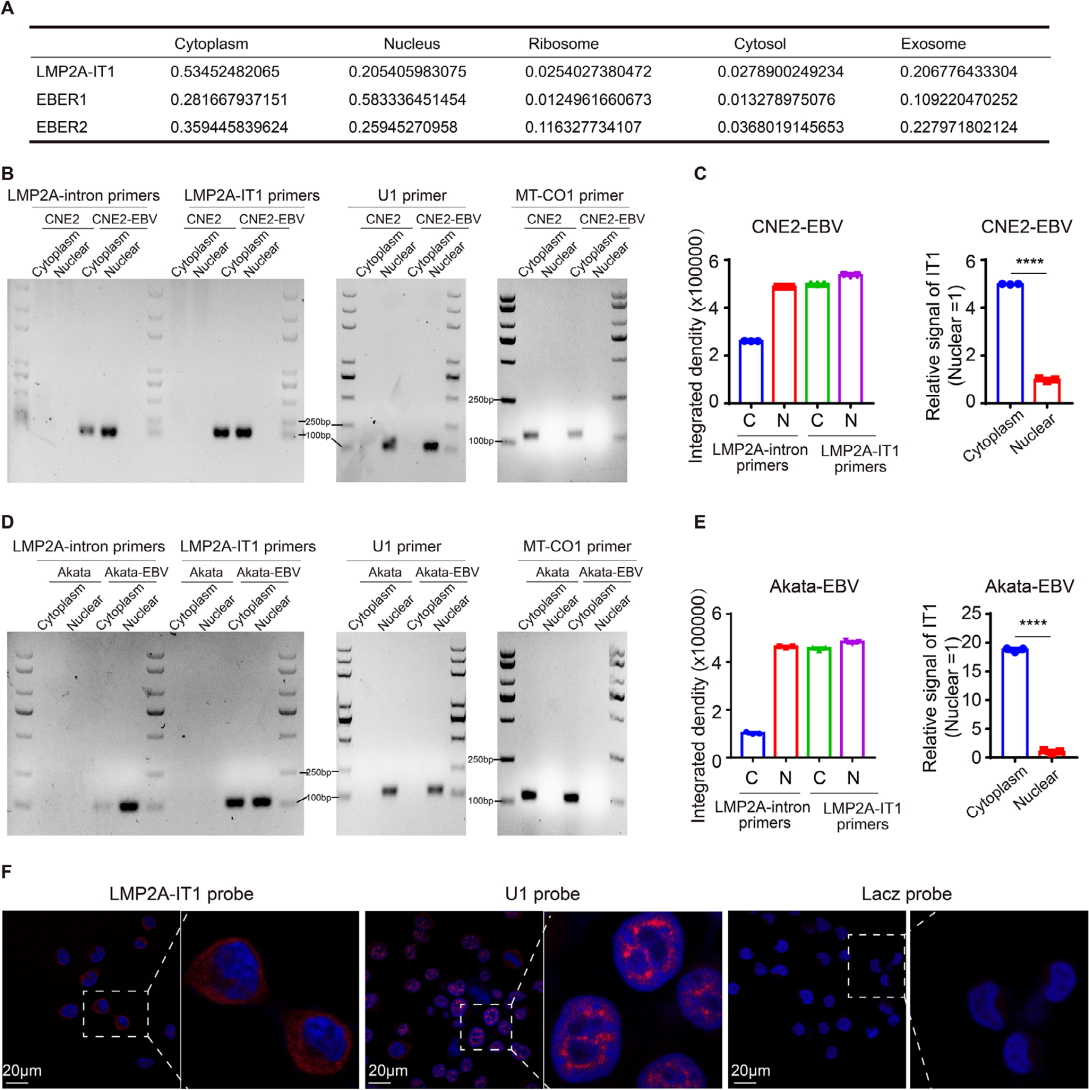
Subcellular localization analysis of LMP2A‐IT1. (A) Prediction of subcellular localization of LMP2A‐IT1 using 1.0 version of lncLocator. (B, C) Subcellular localization analysis in CNE2‐EBV cells. RNA was extracted from cytoplasmic and nuclear fractions following cellular fractionation. First‐strand cDNA was synthesized using either gene‐specific primers (GSP) or random hexamers, followed by PCR amplification with the indicated primers. The amplification products were resolved by agarose gel electrophoresis (B). Band intensities were quantified using ImageJ software (C). The relative signal of LMP2A‐IT1 in the cytoplasm (normalized to the nuclear fraction, defined as 1) is shown in the right panel. C, cytoplasm; N, nucleus. Data are mean ± SD. n = 3 replicates. Comparisons between two groups were performed using two‐tailed unpaired Student's *t*‐test. ^****^ *p* < 0.0001. (D, E) Subcellular localization analysis in Akata‐EBV cells. RNA isolation from fractionated cytoplasmic and nuclear components was performed as in (B), followed by RT‐PCR using the indicated primers. PCR products were resolved by agarose gel electrophoresis (D). Band intensities were quantified using ImageJ software (E). The relative signal of LMP2A‐IT1 in the cytoplasm (normalized to the nuclear fraction, defined as 1) is shown on the right panel. Data are mean ± SD. n = 3. Statistical Analysis was performed by two‐tailed unpaired Student's t‐test. ^****^ *p* < 0.0001. (F) RNA FISH analysis of RNA subcellular localization in CNE2‐EBV cells. Following fixation and permeabilization, the samples were hybridized with biotin‐labelled probes as indicated in the figure. The signals were then detected using cyanine3 (Cy3)‐conjugated streptavidin and visualized under a confocal laser scanning microscope.

The results of gene‑specific RT‑PCR showed that the signal obtained using the LMP2A‐IT1 primers (which amplify both LMP2A‐IT1 and LMP2A pre‑mRNA) was comparable between the nucleus and the cytoplasm, whereas the signal amplified by the LMP2A‑intron primers (which amplify only LMP2A pre‑mRNA) was predominantly nuclear (Figure 3B). Densitometric analysis based on the relative grayscale values further confirmed this observation (Figure 3C, left panel). Using the calculation method described above, we determined that the cytoplasmic level of LMP2A‐IT1 was more than five times the nuclear level (Figure 3C, right panel), indicating that LMP2A‐IT1 is mainly localized in the cytoplasm. We repeated the subcellular localization analysis in EBV‑positive Akata cells (Akata‑EBV), and the results showed that the cytoplasmic level of LMP2A‐IT1 was nearly 20‑fold higher than the nuclear level (Figure 3D,E). Furthermore, we performed RNA fluorescence in situ hybridization (FISH) analysis, which revealed that the signal from the U1 probe was exclusively nuclear while the negative control probe LacZ produced no detectable signal, thereby validating the reliability of our experimental approach. In parallel, the LMP2A‐IT1 probe yielded a signal that was predominantly cytoplasmic (Figure 3F). Collectively, these results demonstrate that LMP2A‐IT1 is a cytoplasmic transcript.

### 2.4 LMP2A‐IT1 is Associated With RNA Spliceosome and Nuclear‐Export Proteins

As the functions of most ncRNAs are mediated by interactions with RNA‐binding proteins, we next identified the proteins that bind LMP2A‐IT1 using an in vitro RNA pull‐down assay (Figure 4A) based on tRNA‐scaffolded streptavidin aptamer (tRSA) adapter [31, 32, 33]. In this experiment, we fused the LMP2A‐IT1 sequence to the 3' end of the tRSA tag to enable enrichment on streptavidin‐coupled beads. To reduce interference from non‐specific binding proteins, we introduced two negative control groups, one containing only the tRSA tag, and the other containing the anti‐sense (AS) strand sequence of LMP2A‐IT1 fused to the 3' end of the tRSA tag. The subsequent in vitro RNA pull‐down assays respectively yielded the binding proteins of tRSA, tRSA‐IT1, and tRSA‐AS, which were resolved by SDS‐PAGE and visualized via silver staining. The results showed a clear specific band at approximately 135 kDa in the tRSA‐IT1 group (Figure 4B), indicating that LMP2A‐IT1 interacts with specific binding proteins. Further mass spectrometry analysis yielded 92 candidate specific interacting proteins that interact with LMP2A‐IT1 (Figure 4C). Among candidates near 135 kDa, the exportin‐5 (XPO5) protein ranked highest in confidence. Therefore, we performed in vitro RNA pull‐down assays followed by western blotting using either whole‐cell lysates (Figure 4D) or purified recombinant XPO5 protein (Figure 4E). In both cases, the results consistently indicated that LMP2A‐IT1 interacts with XPO5.

**FIGURE 4:**
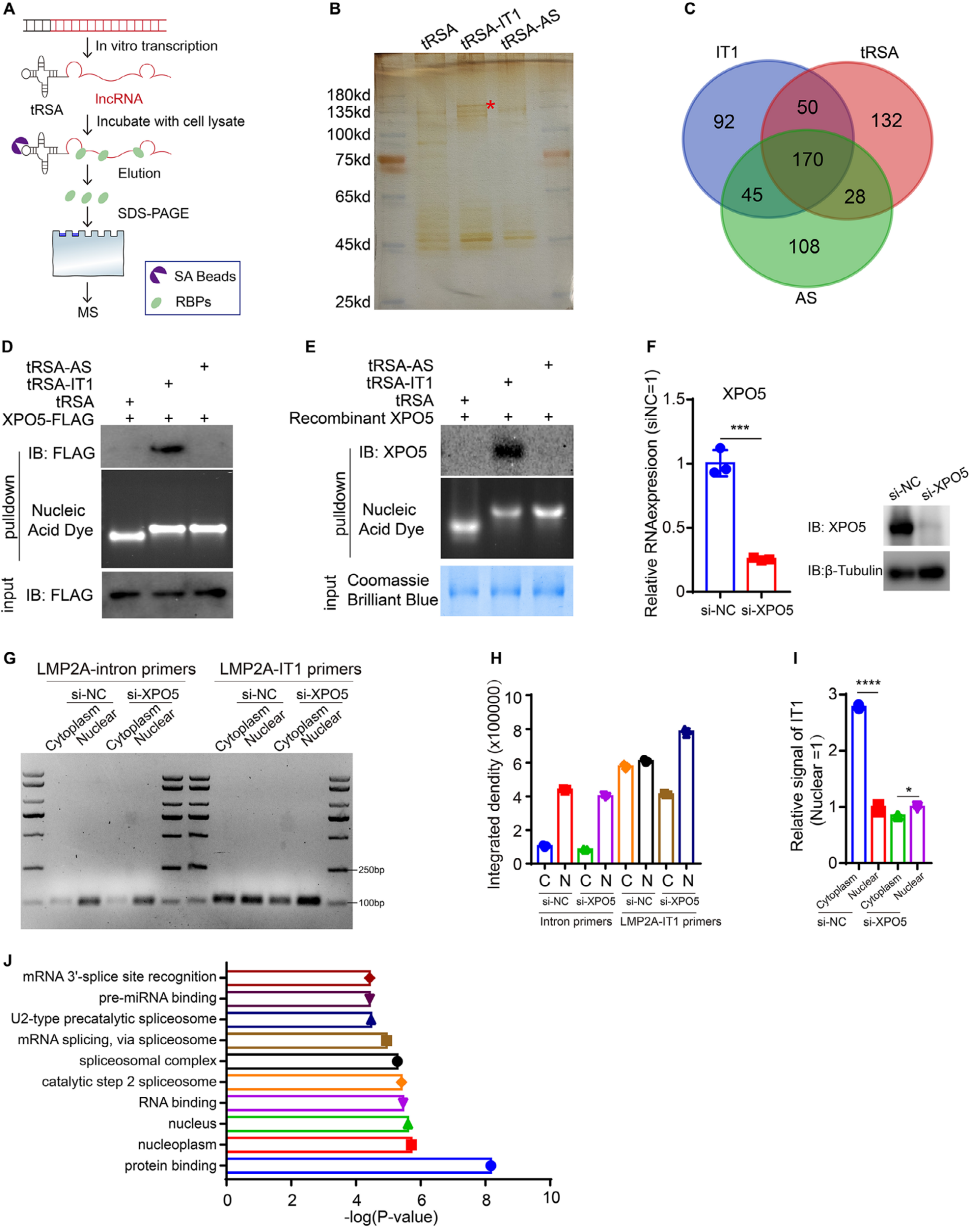
LMP2A‐IT1 is associated with spliceosomes and nuclear‐export proteins. Schematic workflow of the RNA pull‐down assay. The tRSA‐tagged LMP2A‐IT1 (tRSA‐IT1) or control RNAs (tRSA‐AS, tRSA) were prepared by in vitro transcription and immobilized on agarose beads, which were then incubated with CNE2‐EBV cell lysates, followed by elution of captured proteins for SDS‐PAGE or mass spectrometry analysis. SA beads, streptavidin coupled‐ beads; RBPs, RNA binding proteins. The icons in the schematic workflow are sourced from Openclipart (https://openclipart.org/). (B) SDS‐PAGE and silver staining. RNA‐binding proteins were resolved by SDS‐PAGE and visualized by silver staining. (C) Venn diagram analysis of proteins captured by LMP2A‐IT1 versus control RNAs revealing candidate LMP2A‐IT1‐specific binding proteins. (D, E) Validation of the interaction between LMP2A‐IT1 and XPO5. Beads immobilized with LMP2A‐IT1 or control RNAs were incubated with purified recombinant XPO5 protein (E) or lysates from 293T cells transfected with XPO5‐FLAG expression plasmid (D). The eluted proteins were analyzed by western blotting. RNA immobilization was confirmed by on‐beads RT‐PCR and agarose gel electrophoresis. (F) Verification of the knockdown efficiency of si‐XPO5. CNE2‐EBV cells were transfected with si‐NC or si‐XPO5 for 48h, followed by the expression analysis of XPO5 by RT‐qPCR or western blot. Data are mean ± SD. n = 3 replicates. Comparisons between two groups were performed using two‐tailed unpaired Student's t‐test. ^***^ *p* < 0.001. (G–I) Subcellular localization analysis in CNE2‐EBV cells transfected with si‐NC or si‐XPO5. The CNE2‐EBV cells were transfected with si‐NC or si‐XPO5 for 48h, after which RNA was extracted from cytoplasmic and nuclear fractions. First‐strand cDNA was synthesized using gene‐specific primers, followed by PCR amplification with the indicated primers. The amplification products were resolved by agarose gel electrophoresis (G). Band intensities were quantified using ImageJ software (H). The relative signal of LMP2A‐IT1 in the cytoplasm (normalized to the nuclear fraction, defined as 1) is shown in (I). C, cytoplasm; N, nucleus. Data are mean ± SD. n = 3 replicates. One‐way analysis of variance (ANOVA) was carried out with Tukey's correction for multiple comparisons. ^*^ *p* < 0.05; ^****^ *p* < 0.0001. (J) Gene ontology (GO) analysis of 92 candidate specific binding proteins that interact with LMP2A‐IT1.

XPO5 is a widely reported nuclear transporter that transports multiple non‐coding RNAs, including micro‐RNA precursors, deacylated and aminoacylated tRNAs, as well as adenovirus VA1 dsRNA, from the nucleus to the cytoplasm [34, 35, 36, 37]. Thus, we investigated whether XPO5 mediates the nucleus‐to‐cytoplasm shuttling of LMP2A‐IT1. To achieve this, the expression of XPO5 was knocked down using a specific siRNA (Figure 4F) followed by subcellular localization analysis as in Figure 3. Notably, siXPO5‐transfected cells exhibited reduced cytoplasmic localization and concurrent nuclear retention of LMP2A‐IT1 (Figure 4G–I), demonstrating that XPO5 facilitates the nuclear export of LMP2A‐IT1. Gene ontology (GO) analysis of the 92 candidate interacting proteins revealed significant enrichment for components of the RNA splicing machinery, with functions in mRNA recognition and processing (Figure 4J). This result suggests that LMP2A‐IT1 may be involved in RNA splicing, or that the RNA splicing machinery is involved in the maturation of LMP2A‐IT1. Together, these results suggest that LMP2A‐IT1 may originate as a processing byproduct during post‐transcriptional maturation of LMP2A pre‐mRNA, before being exported to the cytoplasm by XPO5.

### 2.5 LMP2A‐IT1 Expression was Significantly Elevated During EBV Lytic Replication

EBV‐encoded products show distinct temporal expression patterns depending on whether the virus is in the latent or lytic phase. To determine in which phase LMP2A‐IT1 is primarily expressed, we induced EBV lytic replication in CNE2‐EBV cells using 12‐O‐tetradecanoylphorbol‐13‐acetate (TPA) and sodium butyrate (NaB), and monitored LMP2A‐IT1 expression. For accurate quantification, we developed a gene‐specific RT‐qPCR approach calibrated using a standard curve. This method allowed us to separately calculate the copy numbers of templates recognized by the LMP2A‐IT1 primers and those recognized by the LMP2A‐intron primers. The true abundance of LMP2A‐IT1 was then obtained by subtracting the signal obtained using the intron‐specific primers from that obtained using the LMP2A‐IT1 primers (Figure S3A). Using this strategy, we found that upon entry of EBV into the lytic replication cycle, as evidenced by greatly increased levels of BZLF1 and BRLF1, the expression of LMP2A‐IT1 gradually rose, reaching a peak of approximately 10‐fold induction in CNE2‐EBV cells (Figure 5A). Similarly, when we triggered lytic reactivation in Akata‐EBV cells with anti‐IgG, successful entry into the lytic replication cycle (again evidenced by elevated BZLF1 and BRLF1) led to a more than 10‐fold increase of LMP2A‐IT1 levels (Figure 5B). By contrast, EBV in B95‐8 cells behaved very differently. Although lytic replication was efficiently induced in these cells (as shown by strong upregulation of BZLF1 and BRLF1), LMP2A‐IT1 did not show a sustained increase. Instead, its levels first dropped sharply and then rebounded only to approximately twice the baseline (Figure 5C). This discrepancy may be attributed to differences between viral strains, as the EBV harbored by CNE2 and Akata cells belongs to the Akata strain, which exhibits considerable sequence divergence from the B95‐8 strain—particularly within the LMP2A‐IT1 locus itself (Figure 2C).

**FIGURE 5:**
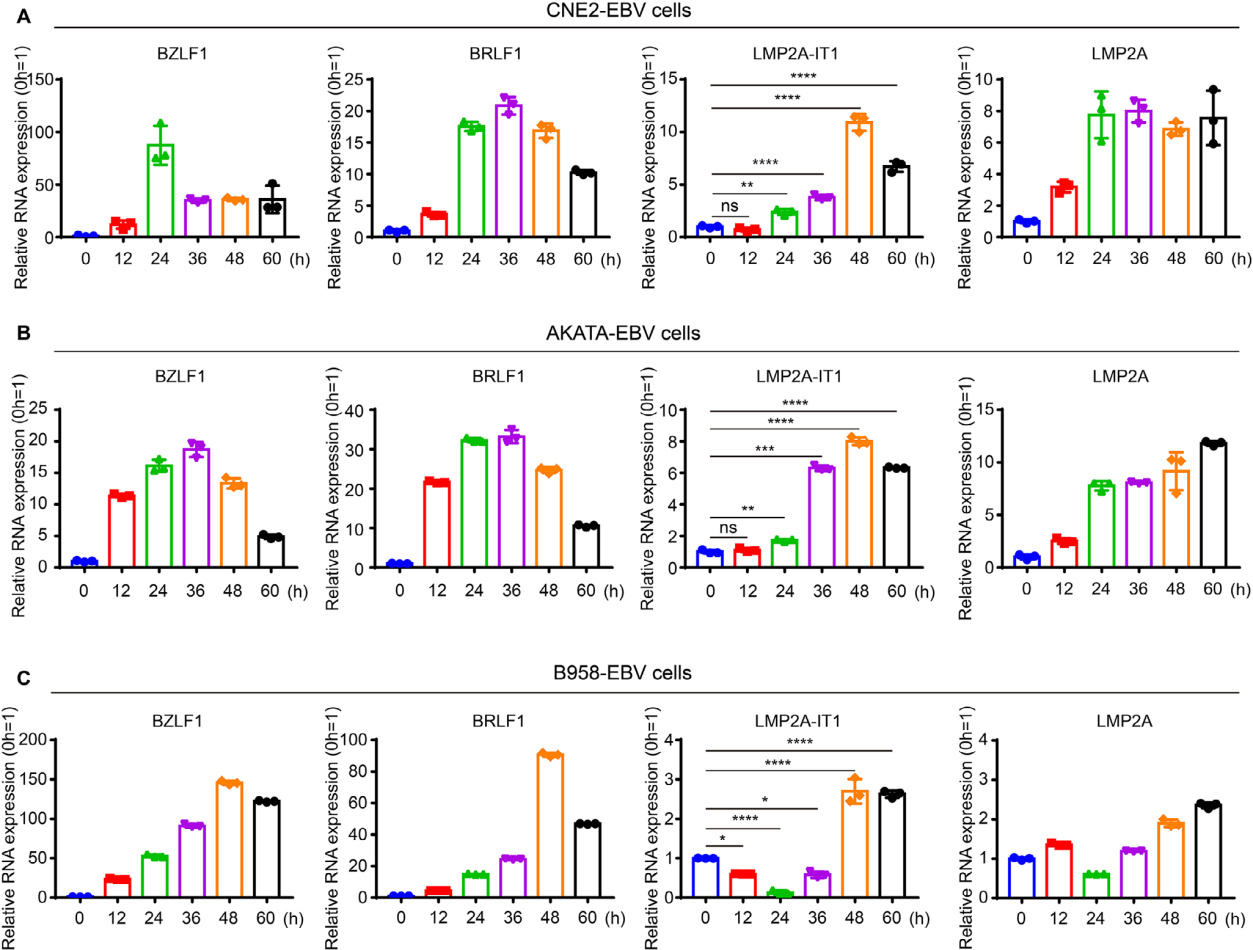
Changes in LMP2A‐IT1 expression during EBV lytic replication. (A)The RNA expression levels of BZLF1, BRLF1, LMP2A and LMP2A‐IT1 during the lytic replication of EBV. CNE2‐EBV cells treated with TPA (20 ng/ml) and NaB (2.5 mM) were collected at the indicated time points, followed by RT‐qPCR analysis of the expression changes of BZLF1, BRLF1, LMP2A and LMP2A‐IT1. Data are mean ± SD. n = 3 replicates. One‐way analysis of variance (ANOVA) was carried out with Dunnett's test for multiple comparisons. ns, no significance; ^**^ *p* < 0.01; ^****^ *p* < 0.0001. (B) Akata‐EBV cells treated with anti‐IgG were collected at the indicated time points, followed by RT‐qPCR analysis of the expression changes of BZLF1, BRLF1, LMP2A and LMP2A‐IT1. Data are mean ± SD. n = 3 replicates. One‐way analysis of variance (ANOVA) was carried out with Dunnett's test for multiple comparisons. ns, no significance; ^**^ *p* < 0.01; ^***^ *p* <  0.001; ^****^ *p* < 0.0001. (C) B95‐8 cells treated with TPA (20 ng/ml) were collected at the indicated time points, followed by RT‐qPCR analysis of the expression changes of BZLF1, BRLF1, LMP2A and LMP2A‐IT1. Data are mean ± SD. n = 3 replicates. One‐way analysis of variance (ANOVA) was carried out with Dunnett's test for multiple comparisons. *p < 0.05; ****p < 0.0001.

Interestingly, when we examined LMP2A mRNA expression using primers targeting its CDS region, we observed a progressive increase during lytic replication of Akata EBV, following a trend very similar to that of LMP2A‐IT1 (Figure 5). This correlation between LMP2A‐IT1 and LMP2A mRNA suggests a potential biosynthetic relationship, which we further address in the Discussion. Taken together, these results demonstrate that LMP2A‐IT1 is robustly upregulated during EBV lytic replication, pointing to an important functional role in the lytic cycle.

### 2.6 LMP2A‐IT1 Promoted EBV Replication by Enhancing the Expression of BZLF1

To functionally characterize LMP2A‐IT1, we engineered a doxycycline (DOX)‐inducible system for LMP2A‐IT1 overexpression in CNE2‐EBV cells. In this cell line, a gradient of LMP2A‐IT1 expression levels could be achieved by induction with different concentrations of DOX (Figure S3B). The RNA sequencing results of cells collected 48 h after DOX induction (Figure 6A,B) as well as the follow‐up qPCR validation (Figure S4A) revealed global upregulation of EBV genes after inducing LMP2A‐IT1 expression, indicating enhanced viral replication and directly implicating LMP2A‐IT1 in promoting lytic cycle progression. This indicates a positive feedback between LMP2A‐IT1 expression and EBV lytic replication, whereby EBV lytic replication drives LMP2A‐IT1 production, and LMP2A‐IT1 in turn promotes lytic replication. Notably, this relationship was further substantiated by further findings in clinical specimens. Specifically, we co‐extracted RNA and DNA from human nasopharyngeal carcinoma tissues and determined the copy numbers of LMP2A‐IT1 and EBV DNA by absolute quantitative PCR. The results showed a significant positive correlation between linear EBV DNA copy number and LMP2A‐IT1 transcript copy number, with a correlation coefficient as high as 0.9049 (Figure 6C). Therefore, LMP2A‐IT1 likely functions as a positive feedback regulator enhancing EBV lytic replication.

**FIGURE 6:**
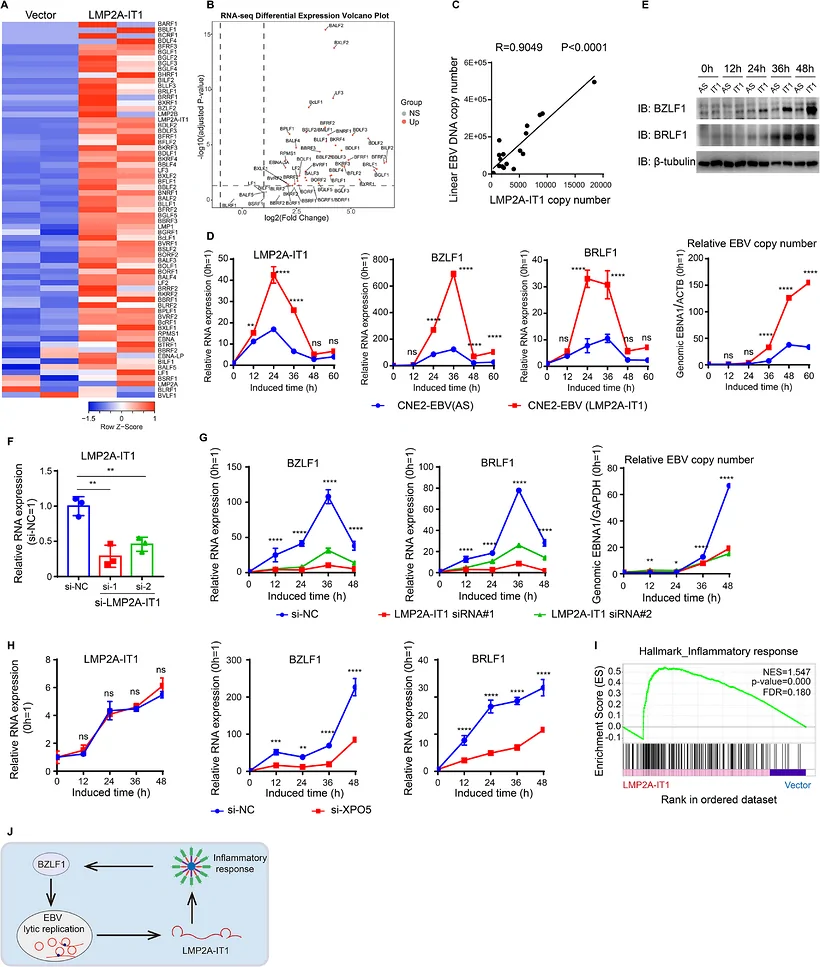
LMP2A‐IT1 promoted EBV lytic replication by upregulating BZLF1. (A, B) Heatmap (A) and volcano plot (B) showing the upregulation of EBV‐encoded genes after induction of LMP2A‐IT1 overexpression with DOX. (C) Correlation between LMP2A‐IT1 transcript copy number and EBV DNA copy number in nasopharyngeal carcinoma tissues. RNA and DNA were co‐extracted from 17 EBV‐positive nasopharyngeal carcinoma tissues, followed by absolute quantitative qPCR analysis of the absolute copy numbers of LMP2A‐IT1 and linear lytic‐phase EBV DNA. Data are mean of technical replicates. n = 3 replicates. The solid line represents the best‐fit linear regression line. Correlation was assessed using Pearson rank correlation coefficient (two‐tailed). *p* < 0.0001. (D) Effect of LMP2A‐IT1 on the expression of BZLF1 and BRLF1. CNE2‐EBV cells harboring the inducible system for LMP2A‐IT1 expression (CNE2‐EBV(LMP2A‐IT1)) or anti‐sense (CNE2‐EBV(AS)) were treated with inducer (DOX + TPA + NaB), and collected at the indicated time points for RT‐qPCR analysis of the expression changes of BZLF1, BRLF1, and LMP2A‐IT1 as well as the relative copy number of intracellular EBV genomic DNA. Data are mean ± SD. n = 3 replicates. Two‐way analysis of variance (ANOVA) was carried out with Sidak's test for multiple comparisons. asterisks (*) indicate statistically significant differences between the indicated groups at the corresponding time points. ns, no significance; ^**^ *p* < 0.01; ^****^ *p* < 0.0001. (E) Cells treated as in (D) were collected at the indicated time points, followed by western blot analysis for the expression of BZLF1 and BRLF1. (F) Validation of LMP2A‐IT1 knockdown efficiency. Following 36h after transfection of CNE2‐EBV cells with siRNA, the total RNA was extracted for RT‐qPCR analysis of the expression level of LMP2A‐IT1. Data are mean ± SD. n = 3 replicates. One‐way analysis of variance (ANOVA) was carried out with Dunnett's test for multiple comparisons. ^**^ *p* < 0.01. (G) Assessment of the impact of LMP2A‐IT1 knockdown on EBV lytic replication. CNE2‐EBV cells were transfected with siRNA for 12 h, followed by treatment with TPA and NaB to induce EBV lytic reactivation. At the indicated time points post‐induction, the cells were harvested for quantification of BZLF1 and BRLF1 expression, as well as EBV DNA copy numbers. Data are mean ± SD. n = 3 replicates. Two‐way analysis of variance (ANOVA) was carried out with Tukey's test for multiple comparisons. asterisks (*) indicate statistically significant differences between the si‐NC and siRNA#2 groups at the corresponding time points. ^*^ *p* < 0.05;^**^ *p* < 0.01;^****^ *p* < 0.0001. (H) Effect of XPO5 knockdown on the expression of BZLF1 and BRLF1. CNE2‐EBV cells transfected with si‐NC or si‐XPO5 for 48h were treated with EBV reactivation inducers (TPA + NaB). The cells were collected at the indicated time points, followed by RT‐qPCR analysis of the expression levels of BZLF1, BRLF1 or LMP2A‐IT1. Data are mean ± SD. n = 3 replicates. Two‐way analysis of variance (ANOVA) was carried out with Sidak's test for multiple comparisons. asterisks (*) indicate statistically significant differences between the indicated groups at the corresponding time points. ns, no significance;^**^ *p* < 0.01;^***^ *p* < 0.001;^****^ *p* < 0.0001. (I) GSEA analysis of RNA‐sequencing data of CNE2‐EBV(LMP2A‐IT1) and CNE2‐EBV(Vector) cells based on hallmark gene sets. NES, normalized enrichment score. FDR, false discovery rate. (J) Schematic diagram of the positive feedback mechanism through which LMP2A‐IT1 promotes EBV lytic replication, with arrows representing promotion.

Given the master regulatory roles of BZLF1 and BRLF1 in EBV reactivation, we hypothesized that LMP2A‐IT1 might promote lytic replication by enhancing the expression of BZLF1 or BRLF1. To verify this hypothesis, we simultaneously treated CNE2‐EBV cells with both DOX (to induce LMP2A‐IT1) and lytic reactivation inducers (TPA and NaB), after which we quantified changes in the expression of BZLF1 and BRLF1 as well as the relative copy number of EBV DNA. As hypothesized, cells overexpressing LMP2A‑IT1 showed sustained elevation of BZLF1 at both the RNA and protein levels throughout the lytic cycle, accompanied by significantly higher EBV genomic copy numbers compared to the anti‐sense (AS) controls (Figure 6D,E). Conversely, in a complementary loss‐of‐function approach, we knocked down LMP2A‐IT1 using siRNA (Figure 6F), which led to global downregulation of EBV gene expression (Figure S4B). Furthermore, after TPA/NaB induction in the siRNA‐transfected cells, we monitored the expression kinetics of BZLF1 and BRLF1 as well as the relative EBV DNA copy numbers. The results revealed that LMP2A‐IT1 depletion suppressed the progressive increase of BZLF1 and BRLF1 expression during the lytic cycle, with peak levels lower than those in the si‐NC‐transfected control group, ultimately resulting in diminished EBV DNA replication (Figure 6G). Taken together, these gain‐ and loss‐of‐function data collectively suggest that LMP2A‐IT1 acts as an enhancer of BZLF1/BRLF1‐driven lytic replication.

Since the XPO5 protein directly interacts with LMP2A‐IT1 and is responsible for mediating LMP2A‐IT1 transfer from the nucleus to the cytoplasm, we investigated whether XPO5 consequently influences the ability of LMP2A‐IT1 to promote EBV lytic replication. Following transfection of CNE2‐EBV cells with si‐XPO5, we induced EBV lytic reactivation and monitored changes of BZLF1 and BRLF1 expression over time. The results showed that XPO5 knockdown significantly attenuated BZLF1 and BRLF1 upregulation during lytic replication, while the LMP2A‐IT1 upregulation trend remained unchanged (Figure 6H). This suggests that the interaction between XPO5 and LMP2A‐IT1 not only affects the subcellular localization of LMP2A‐IT1 but also impacts its functional activity. Collectively, these findings confirm that LMP2A‐IT1 is a key viral factor regulating BZLF1 expression and underscore the functional significance of its cytoplasmic localization.

Further Gene Set Enrichment Analysis (GSEA) of host genes identified significant activation of inflammatory response pathways in LMP2A‐IT1‐overexpressing cells (Figure 6I). We verified these changes in the expression of genes related to the inflammatory response by qPCR, and the results showed that most of the genes (including the classical inflammatory cytokine IL10) were significantly upregulated, using either the empty vector or antisense (AS) as the control (Figure S4C,D). Correspondingly, knockdown of LMP2A‐IT1 by siRNA led to the downregulation of these inflammatory genes (Figure S4E), reflecting the specific role of LMP2A‐IT1 in activating the inflammatory response. Notably, prior studies found that inflammasome activation can relieve the inhibitory effect of the epigenetic repressor KAP1 on the BZLF1 gene, leading to the upregulation of BZLF1 expression and activation of EBV lytic replication [38, 39]. Mechanistically, LMP2A‐IT1 may therefore amplify the expression of BZLF1 by activating the inflammatory cascade to potentiates EBV lytic replication (Figure 6J).

## 3 Discussion

Sixty years after its discovery, Epstein‐Barr virus (EBV) continues to reveal profound complexities. While significant progress has been made in elucidating key aspects of its infection process, pathogenesis, and therapeutic interventions, fundamental gaps persist in our understanding of the intricate latency‐lytic switch as well as the functional repertoire of uncharacterized viral proteins and RNAs [40, 41]. Within its 170‐kb genome, numerous unannotated sequences remain as functional “dark matter” potentially orchestrating critical yet unrecognized aspects of EBV biology. Here, we identified LMP2A‐IT1 as a novel EBV transcript. This study revealed that it is markedly upregulated during EBV lytic replication and forms part of a self‐reinforcing positive feedback loop, illustrating a sophisticated evolutionary strategy for optimizing viral propagation.

Looking back at the research journey, the discovery of LMP2A‐IT1 owes much to serendipity. Despite a number of previous transcriptome‐wide studies of EBV [42, 43, 44, 45], this transcript had never been reported, which may be attributed to its extremely low abundance in the latent phase. As our sequencing results showed, the reads of LMP2A‐IT1 were also scarce in the input sample (Figure 1E–H). However, a novel method for the enrichment of glycoRNAs [21] enabled us to discover this transcript in our sequencing data. Although glycoRNA sequencing reads also mapped to other regions of the EBV genome (Figure 1E), the highest read counts were those aligned with the LMP2A‐IT1 transcript, indicating that it constitutes the main EBV‐derived glycoRNA. Thus, our findings not only identify a previously unknown EBV RNA but to our best knowledge, also provide the first evidence that viral RNAs can be glycosylated. However, technical limitations and the lack of established approaches for studying the function of an individual glycoRNA prevented us from further characterizing the glycosylated form of LMP2A‐IT1. Whether glycosylation affects its subcellular localization, protein interactions, or biological function remains an important question for future studies.

It should be noted that the biogenesis of LMP2A‐IT1 remains incompletely understood. Its strong positive correlation with LMP2A mRNA expression (Figure 5) suggests it may be transcribed from the same promoter. However, LMP2A‐IT1 lacks key transcriptional hallmarks of RNA polymerase II, such as a 5' cap and a poly(A) tail, making it highly unlikely to be transcribed independently and is much more likely generated through the splicing/processing of LMP2A pre‐mRNA. Indeed, our in vitro RNA pull‐down results indicate that LMP2A‐IT1 interacts with host splicing factors and the key RNA export protein XPO5 (Figure 4). Furthermore, subcellular fractionation assays showed that LMP2A‐IT1 is primarily localized to the cytoplasm (Figure 3). Collectively, these findings point toward a potential biogenesis model whereby host nuclear splicing factors bind to the first intron during the post‐transcriptional processing of LMP2A pre‐mRNA to excise LMP2A‐IT1, which is subsequently exported to the cytoplasm by XPO5 to exert its function. This hypothesis warrants further investigation in future studies.

We established that LMP2A‐IT1 enhances EBV lytic replication by activating inflammatory responses that boost BZLF1 expression. However, the mechanism underlying inflammasome activation remains unclear. We found that LMP2A‐IT1 contains an open reading frame (ORF) with the capacity to potentially encode a 25‐amino‐acid micropeptide (Figure S5). However, although we synthesized the corresponding peptide and generated an antibody against it, repeated attempts to detect the endogenous peptide via western blotting and mass spectrometry failed, likely due to its low abundance and/or high instability when dissociated from the intracellular microenvironment. Despite this, the coding potential of LMP2A‐IT1 introduces substantial complexity to its functional mechanism. Critically, since both viral RNAs and proteins/peptides can activate inflammasomes, determining whether LMP2A‐IT1 functions as an RNA molecule or through its encoded micropeptide is essential for understanding its pro‐lytic role.

Although we have uncovered a role for LMP2A‐IT1 in promoting EBV lytic replication, we cannot exclude the possibility that this viral RNA may exert additional functions beyond the characterized activity. In Figure 4, we have already performed RNA pull‐down assays using in vitro transcribed LMP2A‐IT1 to identify its interacting protein partners. However, the major interactors we identified were predominantly nuclear export proteins and components of the RNA splicing/processing machinery, neither of which directly participates in the functional effects we observed. The failure of this approach to yield functionally relevant interactors may be attributable to the fact that the in vitro transcribed RNA lacks glycosylation modifications or may fail to properly fold into its native conformation, potentially leading to the loss of some physiologically relevant binding partners. Future efforts aimed at identifying LMP2A‐IT1 interacting proteins under native cellular conditions, or alternative functional screening strategies, may uncover additional activities of this non‐coding RNA.

In summary, we identified LMP2A‐IT1 as a novel EBV‐encoded RNA and discovered that it is subjected to glycosylation. We systematically characterized its full‐length sequence, subcellular localization, interacting proteins, and expression dynamics throughout the EBV lytic cycle. These investigations revealed its functional role in promoting EBV lytic replication through a positive feedback mechanism. These findings significantly advance our understanding of the EBV genome while highlighting that a considerable number of unannotated sequences remain to be more deeply investigated, as they may exert a subtle yet profound influences on EBV biology akin to genomic “dark matter”. Further investigations will substantially deepen our understanding of this novel RNA with significant potential for the discovery of new therapeutic targets and diagnostic biomarkers for EBV‐associated diseases.

## 4 Materials and Methods

### 4.1 Sample Collection

The collection and use of human nasopharyngeal carcinoma tissue samples for our research was approved by the Ethics Committee of Sun Yat‐sen University Cancer Center (Approval no. G2024‐171‐X01). The tissue samples were originally obtained through standard clinically indicated procedures during routine diagnosis and treatment, and had been stored in the Biobank of Sun Yat‐sen University Cancer Center prior to the commencement of this research. As the original participants were no longer contactable, a formal waiver of informed consent for the use of these archived tissues was granted by the ethics committee. We confirm that all samples were fully de‐identified, with all demographic and health information removed, and no potentially identifiable genetic information was collected during the study.

### 4.2 Cell Culture

HEK‐293T cells (ATCC, CRL‐3216) were cultured in Dulbecco's modified essential medium supplemented with 10% fetal bovine serum (FBS; FSP500, ExCell Bio) and penicillin/streptomycin. EBV‐positive Akata cells (Akata‐EBV) were a kind gift from professor Maria G. Masucci (Karolinska Institute, Sweden) and cultured in RPMI1640 medium supplemented with 5% FBS (10099‐141C, GIBCO) and penicillin/streptomycin. EBV‐positive CNE2 cells [46] were cultured in RPMI1640 medium supplemented with 5% FBS and penicillin/streptomycin. B95‐8 cells were purchased from Baidi Biotech Ltd (C5033) and cultured in RPMI1640 medium supplemented with 5% FBS and penicillin/streptomycin. All cells were cultured at 37°C in a humidified atmosphere comprising 5% CO_2_.

### 4.3 Plasmids

To construct plasmids with DOX‐inducible expression, the coding sequence of LMP2A‐IT1 was PCR‐amplified from genomic DNA of EBV and then integrated into the pLVX‐TetOne‐Puro vector. For the XPO5 expressing plasmid, the full‐length cDNA sequences of XPO5 was PCR‐amplified and then integrated into the PCR3.1 vector, resulting in a genetic fusion with a C‐terminal 3×FLAG tag. To construct the DNA standards of LMP2A‐IT1 and LMP2A pre‐mRNA for determining their absolute abundance via qPCR, LMP2A‐IT1 and the first 1000bp of the first intron of the LMP2A gene were amplified by PCR and cloned into the PLKO.1 vector. The standard plasmid encoding the EBNA1 gene was a kind gift by professor Kuang Ersheng (Sun Yat‐sen university, China) [47].

### 4.4 RACE Assay

For 3' rapid amplification of cDNA ends (RACE) analysis, RNA from CNE2‐EBV cells was initially dephosphorylated using T4 polynucleotide kinase (D7099M, Beyotime Biotechnology) to remove 3'‐phosphate groups. The dephosphorylated RNA was then polyadenylated using *E. coli* poly(A) polymerase (R7070M, Beyotime Biotechnology). First‐strand cDNA synthesis was then performed using a 3RACE‐adaptor primer and M‐MLV reverse transcriptase (D7160M, Beyotime Biotechnology). The first round PCR amplification was carried out using the resulting cDNA as template with the 3RACE outer primer and gene‐specific primer (3RACE‐GSP1). The second round nested PCR was subsequently performed using the first round PCR products as template with the 3RACE inner primer and nested gene‐specific primer (3RACE‐GSP2). The second round PCR products were cloned into the pCR3.1 vector and verified by Sanger sequencing. For 5' RACE analysis, RNA from CNE2‐EBV cells was reverse‐transcribed using a gene‐specific primer (5RACE‐RT‐GSP). The cDNA was purified to remove unincorporated primers and dNTPs, followed by polyadenylation of the 3' end using terminal deoxynucleotidyl transferase (EP0161, Thermo Scientific). Second‐strand cDNA synthesis was subsequently performed using a 5RACE adaptor primer. The first round PCR amplification was conducted using the double‐stranded cDNA template with the 5RACE outer primer and gene‐specific primer (5RACE‐GSP1). The second round nested PCR amplification employed first round PCR products as template with the 5RACE inner primer and nested gene‐specific primer (5RACE‐GSP2). The final PCR products were cloned into the pCR3.1 vector and confirmed by Sanger sequencing.

### 4.5 Nuclear‐Cytoplasmic Fractionation for Subcellular RNA Extraction

Total cellular RNA was fractionated into nuclear and cytoplasmic components using the PARIS Kit (AM1921, Life Technologies) according to the manufacturer's protocol. Specifically, the CNE2‐EBV cells or Akata‐EBV cells were collected, washed with ice‐cold PBS, and maintained on ice. The cell pellet was resuspended in 500 µL ice‐cold Cell Fractionation Buffer and incubated on ice for 5–10 min. After centrifugation at 500 for 5 min at 4°C, the supernatant (cytoplasmic fraction) was carefully aspirated from the pellet (nuclear fraction). The pellet was washed once with ice‐cold Cell Fractionation Buffer and resuspended in 500 µL of ice‐cold Cell Disruption Buffer. For RNA isolation, the cytoplasmic fraction and nuclear lysate were each mixed with an equal volume of 2x Lysis/Binding Solution at room temperature, followed by the addition of one sample volume of 100% ethanol. The sample mixture was then drawn through the provided Filter Cartridge, which was washed once with 700 µL Wash Solution 1 and twice with 500 µL Wash Solution 2/3. Finally, the RNA was eluted with 40 µL of preheated Elution Solution.

### 4.6 Gene‐Specific RT‐PCR and Subcellular Localization Analysis of LMP2A‐IT1

Following RNA extraction from cytoplasmic and nuclear fractions, 2 µg of RNA from each compartment was reverse‐transcribed using the gene‐specific primer (IT1‐RT‐GSP). The resulting cDNA served as template for parallel PCR amplifications with two primer pairs: 1) the LMP2A‐intron primers specifically amplifying precursor transcripts (i.e., the pre‐mRNA of LMP2A), and 2) LMP2A‐IT1 primers amplifying precursor transcripts and the mature LMP2A‐IT1 transcript. The resulting amplification products were resolved by agarose gel electrophoresis and visualized using a Gel Doc XR+ Gel Documentation System. Band intensities were quantified densitometrically using ImageJ software, whereby the specific signal intensity of LMP2A‐IT1 was determined by subtracting the background signal (obtained with LMP2A‐intron primers) from the total signal (obtained with LMP2A‐IT1 primers) for each cellular compartment. Nuclear signal intensity was normalized to 1 to calculate relative cytoplasmic enrichment. Final data visualization and analysis were performed using GraphPad Prism 7 (GraphPad Software Inc., USA).

### 4.7 In Vitro Transcription of RNA

In vitro transcription was performed using the HiScribe T7 Quick High Yield RNA Synthesis Kit (E2050S, New England Biolabs) according to the manufacturer's protocol. Briefly, we constructed clone vectors harboring LMP2A‐IT1 or its anti‐P0017Ssense strand (AS) fused to a 5'‐terminal tRSA adapter sequence. These vectors served as templates for PCR amplification using T7 promoter‐containing primers to generate DNA templates for in vitro transcription. Transcription reaction mixtures were then prepared following the kit protocol and incubated overnight at 37°C. Finally, residual DNA was removed using DNase I (D7076, Beyotime Biotechnology), after which the transcribed RNA was purified, aliquoted, and stored at −80°C.

### 4.8 RNA pulldown

Prior to the pull‐down assay, the in vitro transcribed RNA was subjected to denaturation followed by renaturation to facilitate proper secondary structure formation. Briefly, 3 µg of RNA was heated at 90°C for 2 min, rapidly cooled on ice for 2 min, and refolded in RNA structure buffer (10 mM Tris‐HCl pH 7.0, 0.1 M KCl, 10 mM MgCl_2_) over 20 min at room temperature. The refolded RNA was incubated with streptavidin (SA)‐conjugated beads (P2159, Beyotime Biotechnology) at room temperature for 30 min to enable RNA immobilization. CNE2‐EBV cell lysates prepared in RIP buffer (50 mM Tris‐HCl pH 7.4, 150 mM NaCl, 1 mM MgCl2, 1% NP‐40, 1 mM dithiothreitol, 100U/mL recombinant RNase inhibitor, proteinase inhibitor cocktail) or Recombinant Human XPO5 Protein (IPODIX Biotech Inc, PA1000‐9226) were then added to the RNA‐loaded beads and incubated at room temperature for 1 h on a rotating platform. The beads were then washed five times with RIP buffer to remove non‐specifically bound proteins. Finally, the beads were divided into two aliquots one of which underwent on‐bead reverse transcription followed by PCR detection, while the other was eluted with 5x SDS sample buffer for western blotting or mass spectrometry analysis.

### 4.9 Silver Staining and Mass Spectrometry

Silver staining was performed using the Fast Silver Stain Kit (P0017S, Beyotime Biotechnology) according to the manufacturer's protocol. Mass spectrometry analysis was conducted by Sangon Biotech (Shanghai, China). Briefly, enzymatically digested peptide samples were centrifugally dried, redissolved in mobile phase A (0.1% formic acid in water) for Nano‐LC, and subjected to on‐line LC‐MS/MS analysis. Raw mass spectrometry data (wiff files) were processed in ProteoWizard (v3.0.10577, 64‐bit) for conversion into mgf format. The resulting mgf files were analyzed using the Mascot search engine (v2.3.02) for protein identification.

### 4.10 Gene‐Specific RT‐qPCR

Gene‐specific RT‐qPCR was analogous our gene‐specific RT‐PCR protocol. Briefly, RNA was reverse transcribed using a gene‐specific primer (IT1‐RT‐GSP), after which the resulting cDNA and corresponding plasmid standards are used as templates for qPCR with LMP2A‐intron primers and LMP2A‐IT1 primers. Standard curves were plotted based on the standards, and the copy numbers determined using the two pairs of primers were calculated. The copy number obtained using LMP2A‐IT1 primers minus the copy number obtained using LMP2A‐intron primers is the true copy number of the LMP2A‐IT1 transcript.

### 4.11 Conventional RT‐qPCR

The conventional RT‐qPCR protocol was described our previous study [48]. Briefly, total RNA was isolated using TRIzol reagent (15596018, Thermo Fisher Scientific) or NcmSpin Rapid RNA Extraction Kit (M5106, NCM Biotech, China). For gene expression analyses, 2 µg of RNA was reverse‐transcribed using random primers in conjunction with Color Reverse Transcription Kit (A0010CG, EZBioscience) in a 20 µl reaction mixture. The mRNA level was evaluated by qPCR using the 2x EZ Color SYBR Green qPCR Master Mix (CQ22, EZBioscience) and analyzed on a CFX96 Touch Real‐Time PCR Detection System. All gene expressions levels were normalized to the housekeeping genes GAPDH or ACTB.

### 4.12 RNA Blotting

RNA blotting was performed as previously described [22]. Briefly, cells were first treated with Ac4ManNAz for 48 h, after which RNA was extracted from the cells or exosomes. The RNA samples were then treated with DNase I and proteinase K to remove DNA and protein contaminants, respectively. Subsequently, the RNA was subjected to a click chemistry reaction with DBCO‐PEG4‐biotin reagent in a dye‐free gel loading buffer II system. The labeled RNA samples were resolved on a 1% agarose‐formaldehyde denaturing gel. After electrophoresis, the gel was stained with ethidium bromide, and the staining pattern was documented using a Gel Doc XR+ Gel Documentation System. The RNA was then transferred from the gel to a nitrocellulose membrane by capillary transfer and cross‐linked to the membrane by UV irradiation. Finally, the biotinylated RNA was detected using streptavidin‐horseradish peroxidase (HRP).

### 4.13 RNA Sequencing

GlycoRNA sequencing was performed as described previously [22]. Briefly, total RNA was extracted from Ac4ManNAz‑treated cells, their derived exosomes, and PDX tissues. The RNA samples were then digested with DNase I and proteinase K to respectively remove DNA and proteins, followed by a click chemistry reaction to label glycoRNAs with biotin. Subsequently, RNA fragments shorter than 2000 nucleotides were collected by sucrose density gradient centrifugation. From this preparation, 150 ng of RNA was taken as the input sample, and another 25 µg of RNA was incubated with streptavidin magnetic beads to obtain the enriched glycoRNA fraction. Strand‑specific libraries were then constructed using the VAHTS Universal V6 RNA‑seq Library Prep Kit (Vazyme Biotech, Nanjing, China) for both input and enriched samples. The libraries were sequenced on an Illumina NovaSeq 3000/4000 platform to generate paired‑end 150‑bp reads. To detect cellular transcriptome profiling changes caused by LMP2A‐IT1 overexpression, CNE2‐EBV (LMP2A‐IT1) and CNE2‐EBV (vector) cells were collected to construct rRNA‐depleted strand‐specific RNA libraries, which were subjected to NGS by Berry Genomics Co., Ltd. Raw sequencing data were evaluated using fastp to assess sequence quality metrics. Adapters and low‐quality bases were removed to generate clean reads for downstream analysis. We annotated the location of LMP2A‐IT1 in the EBV genome (Akata strain, accession no. KC207813.1) and integrated the modified EBV genome with the human hg38 genome to generate a combined reference genome for read alignment. Clean reads were aligned to the reference genome using HISAT2 v2.2.1 with the parameter –rna‐strandness RF to preserve strand‐specific information. Aligned reads were assigned to genes using featureCounts v2.0.1 with the parameter ‐s 2 (specific to RF‐stranded libraries). We only counted uniquely mapped reads. Raw count data were imported into the R environment and normalized using DESeq2 v1.34.0. Transcripts Per Million (TPM) values were calculated to estimate gene expression levels.

### 4.14 Sialic Acid Aptamer and RNA In Situ Hybridization‐Mediated Proximity Ligation Assay (ARPLA)

The ARPLA assay was carried out strictly in accordance with a previously established protocol [27], including all buffers and solution compositions described therein. The procedure generally comprised three sequential stages: (i) RNA probe hybridization, (ii) aptamer‐mediated recognition of glycoRNA‐associated Neu5Ac coupled with proximity‐assisted in situ ligation, and (iii) rolling circle amplification (RCA)‐based signal detection. Briefly, CNE2‐EBV cells were seeded onto 35‐mm confocal dishes. After 24 h, the cells were washed three times with PBS and fixed with 4% paraformaldehyde (PFA) at 37°C for 15 min. The cells were then blocked with blocking buffer at 37°C for 30 min, followed by incubation with 1.5 µM RNA‐binding probe targeting LMP2A‐IT1 in hybridization buffer at 37°C for 30 min. The cells were subsequently washed with washing buffer and rinsed with PBS to remove residual formamide. For aptamer recognition and proximity‐assisted ligation, the cells were incubated with the aptamer and connector solution at 37°C for 30 min. Then, the ligation mixture containing T4 DNA ligase (NEB, M0202L) and ATP was added and mixed thoroughly, and the reaction was allowed to proceed in the appropriate ligase buffer. For in situ RCA, the cells were incubated with RCA working solution (containing 2.5U/µl phi29 DNA polymerase (Beyotime, D7053L), dNTPs, and BSA) at 37°C for 90 min. The single‐stranded RCA products were then detected by in situ hybridization with Alexa Fluor 647‐labeled DNA reporter probe at 37°C for 30 min. Finally, the RCA solution was removed, and the cells were mounted with an antifade reagent containing DAPI (Abbkine, BMU107‐10mL). Images were acquired using a Zeiss LSM880 confocal microscope.

### 4.15 RNA FISH

To visualize LMP2A‐IT1 RNA, biotin‐labeled DNA oligonucleotide probes were used. CNE2‐EBV cells were seeded on 35‐mm confocal dishes and cultured to approximately 50% confluence. The cells were washed three times with PBS, fixed with 4% paraformaldehyde for 15 min at room temperature, and permeabilized with ice‐cold 0.5% Triton X‐100 for 8 min at 4°C, followed by additional PBS washes. The cells were then blocked with prehybridization buffer (4×SSC buffer containing 50% formamide and 100 nM poly(T) oligonucleotides) at 37°C for 30 min. Hybridization was performed overnight at 37°C in hybridization buffer composed of 50% formamide, 5×SSC buffer, 10% dextran sulfate, 0.1% SDS, and 5×Denhardt‘s solution, with 1.5 µM of the biotinylated probe targeting LMP2A‐IT1. The 100× Denhardt's stock solution was prepared by dissolving 1 g each of Ficoll, polyvinylpyrrolidone (PVP), and bovine serum albumin (BSA) in 50 mL DEPC‐treated water (resulting in 2% w/v of each component), and was diluted to 5× for use in the hybridization buffer. After hybridization, the cells were sequentially washed with 4×, 2×, and 1× SSC buffer, with three, two, and one wash, respectively, and then rinsed briefly with PBS at room temperature. For signal detection, the cells were incubated with Cy3‐conjugated streptavidin (Solarbio, K1068R‐Cy3) in PBS for 15 min at 4°C in the dark. After three PBS washes, the cells were mounted with an antifade reagent containing DAPI (Abbkine, BMU107‐10mL). Fluorescence images were acquired using a Zeiss LSM880 confocal microscope.

### 4.16 Western Blotting

Cells were lysed on ice for 30 min in lysis buffer (50 mM Tris‐HCl, pH 7.4; 1 mM EDTA, pH 8.0; 10% glycerol; 3% Sodium Dodecyl Sulfate) containing 1x protease inhibitor cocktail (C0001, TargetMol Chemicals Inc) and then centrifuged in 12 000 g for 10 min. The protein concentration of the lysates was measured using a BCA protein assay kit and were normalized to equal amounts of protein. The proteins were resolved on SDS‐PAGE and transferred onto PVDF membranes. The membranes were blocked in 5% skim milk in 1xPBS for 1 h, and then incubated with appropriately diluted primary antibodies overnight at 4°C. The BZLF1 antibody (sc53904) was purchased from Santa Cruz Biotechnology; the BRLF1 antibody (bs‐4542R) was from Bioss Ltd, respectively; the β‐tubulin antibody (2146S) was from Cell Signaling Technology; the Flag antibody (66008‐1‐Ig) was from Proteintech Group, Inc; the XPO5 antibody (CY8731) was from Shanghai Ebiwei Biotechnology Co., Ltd. Finally, the membranes were incubated with species‐specific HRP‐conjugated secondary antibodies and visualizes using a ChemiDoc Touch detection system (BIO‐RAD).

### 4.17 Genomic DNA Extraction From Cultured Cell Lines

To determine the intracellular EBV DNA copy number, CNE2‐EBV cells were collected, and then DNA was extracted using the E.Z.N.A. Tissue DNA Kit (D3396‐02, Omega) according to the product manual.

### 4.18 DNA and RNA Extraction From Nasopharyngeal Carcinoma Tissues

DNA and RNA were co‐extracted from identical nasopharyngeal carcinoma tissue specimens using TRIzol reagent. Tissue samples were first homogenized in TRIzol using a Precellys Evolution Touch homogenizer, then mixed with chloroform, vigorously vortexed, and incubated at room temperature for 5 min. Following centrifugation at 12 000 g for 15 min at 4°C, the aqueous phase was transferred for RNA precipitation with isopropanol, while the interphase and organic phase were retained for DNA isolation. For DNA extraction, 0.3 ml 100% ethanol per 1 ml of the initial TRIzol volume was added to the interphase and organic phase with repeated inversion. After 2‐3 min of incubation at room temperature, DNA was pelleted by centrifugation at 2000 g for 5 min (4°C). The DNA pellet underwent two washes with 0.1 M sodium citrate in 10% ethanol, followed by resuspension in 75% ethanol and final centrifugation at 2000 g for 5 min (4°C). Air‐dried DNA pellets were dissolved in 8 mM NaOH, and the pH of the samples was adjusted to 8.4 with 0.1 M HEPES prior to qPCR analysis.

### 4.19 Quantification of EBV DNA Copy Numbers

Following extraction from cultured cells or nasopharyngeal carcinoma (NPC) tissue samples, the DNA concentration was measured using a NanoDrop One spectrophotometer and adjusted to a uniform concentration across all samples prior to qPCR analysis. For the quantification of EBV genomic DNA in cultured cells, a relative quantification approach was employed. Briefly, qPCR was performed using primers specific to the EBV EBNA1 gene to obtain the quantification cycle (Cq) value for viral DNA. In parallel, primers targeting the host ACTB or GAPDH gene were used to determine the corresponding Cq value for the endogenous reference. The relative EBV DNA level was then calculated as the expression ratio of EBNA1 to ACTB or GAPDH. For absolute quantification of lytic linear EBV DNA in NPC tissue samples, a Plasmid‐Safe ATP‐Dependent DNase (PSAD)‐based qPCR assay was employed. Each DNA sample was divided into two equal aliquots: one was treated with PSAD (Lucigen, E3101K) according to the manufacturer's protocol at 37°C for 12 h, followed by heat inactivation at 70°C for 30 min; the other aliquot was subjected to the same temperature regimen without PSAD addition. Both aliquots were then subjected to qPCR in parallel with a standard curve generated from serial dilutions of an EBNA1 plasmid standard. The copy numbers of EBV DNA in each aliquot were calculated from the standard curve. The PSAD‐treated aliquot represented the circular form of EBV DNA (since the enzyme selectively degrades linear DNA), while the untreated aliquot represented total EBV DNA (both circular and linear). The copy number of linear EBV DNA was thus calculated by subtracting the circular copy number from the total.

### 4.20 Statistical Analysis

Statistical Analyses were performed using GraphPad Prism software (v7.0). Results are representative of two or three independent experiments (n = 3). Data are means ± standard deviation (SD). One‐way analysis of variance (ANOVA) or Two‐way ANOVA was carried out for multiple comparisons, Comparison between two groups was performed by two‐tailed unpaired Student's t test. ^*^ *p* < 0.05; ^**^ *p* < 0.01; ^***^ *p* < 0.001; ^****^ *p* < 0.0001; ns, no significance.

## Author Contributions

L.Z.Z., W.J.F. and Z.N. designed the study, conducted most of the experiments, and wrote the article. Y.M.H., Z.L.L., L.Y.T., C.W.J., T.J.K., G.J.W., L.C.B., Z.T., W.Y., and H.C.Y. participated in some of the experiments. Z.Q. helped with sample collection. L.Z.Z., Z.M.S., and L.Y. provided financial support and technical guidance.

## Conflicts of Interest

The authors declare no conflicts of interest.

## Supporting information




**Supporting File 1**: advs78488‐sup‐0001‐SuppMat.pdf.

**Supporting File 2**: advs78488‐sup‐0002‐glycoRNA‐BAM‐files.zip.

## Data Availability

All study data are included in the article and/or supplemental materials. The mass spectrometry raw data have been deposited with the ProteomeXchange Consortium (https://proteomecentral.proteomexchange.org) via the iProX partner repository with the dataset identifier PXD077863, and the processed data have been uploaded as supplementary data Table S1. The siRNA sequence and primer sequences have been uploaded as supplementary data Table S2. The RNA‐seq data related to Figure 1 of this paper have been deposited in the Sequence Read Archive (SRA) database of National Center for Biotechnology Information (SRR37327565, SRR37327564, SRR37327566, SRR37327567, SRR37327568, SRR37327569, SRR37327570, SRR37327571, SRR37327572, SRR37327573, SRR37327574, SRR37327575, SRR37327563, SRR37327562, SRR37327561, SRR37327560, SRR37327559, SRR37327558, SRR40313845, SRR40313846, SRR40313847, SRR40313848). The RNA‐seq data related to Figure 6 have been deposited in the Gene Expression Omnibus (GEO) database of the National Center for Biotechnology Information (GSE320354).
