# Supplementary material for: The Novel Viral Non‐Coding RNA LMP2A‐IT1 Promotes the Lytic Replication of EBV
Source: Adv Sci (Weinh). 2026 Nov 5:e78488. Online ahead of print. doi: 10.1002/advs.78488 (PMC13735861; doi:10.1002/advs.78488)
Supplement: Supplementary file 1 [file ADVS-9999-e78488-s001.pdf]

Supporting Information for

The Novel Viral Non-coding RNA LMP2A-IT1 Promotes the Lytic Replication of EBV

Zheng-Zhou Lu[1* †], Jun-Fei Wu[1*], Nannan Zhu[2*], Ming-Hui Yang[1], Le-Le Zhang[1], Yuan-Tao Liu[1], Wei-Jun Chen[1], Jin-Kun Tang[1], Jia-Wei Ge[1], Chao-Bo Lei[1], Tao Zhang[1], Yu Wang[3], Chao-Yun Huang[4], Qian Zhong[1], Mu-Sheng Zeng[1,5 †], Yan Li[1,4 †].

*These authors contributed equally to this work.

† Correspondence to: Yan Li (liyan1@sysucc.org.cn), Mu-Sheng Zeng (zengmsh@sysucc.org.cn) and Zheng-Zhou Lu (luzz@sysucc.org.cn)

This Supporting Information file includes:

Fig S1 to S5

Table S1. Candidate binding proteins of LMP2A-IT1 (part a and part b)

Table S2. Primer sequence

Full scans for Fig 1,2,3,4,6.

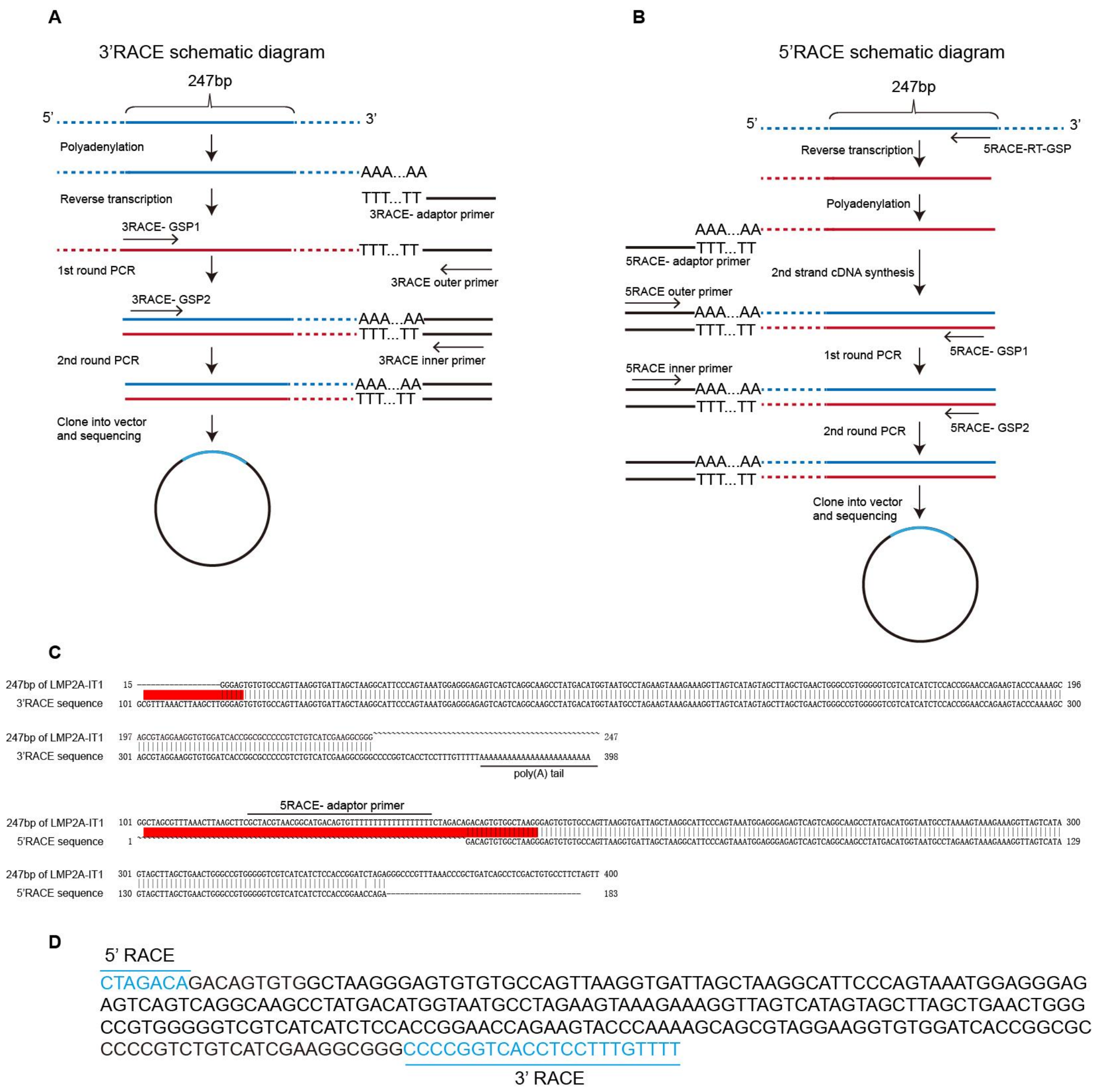

Fig S1. The RACE assay for analyzing the full-length sequence of LMP2A-IT1.

(A, B) Flowchart of the RACE assay. The specific protocols are presented in the MATERIALS AND METHODS.

(C) Sanger sequencing results of amplified products from RACE assay.

(D) The full-length sequence of LMP2A-IT1, the sequence marked in blue is the flanking sequence obtained by RACE experimental amplification.

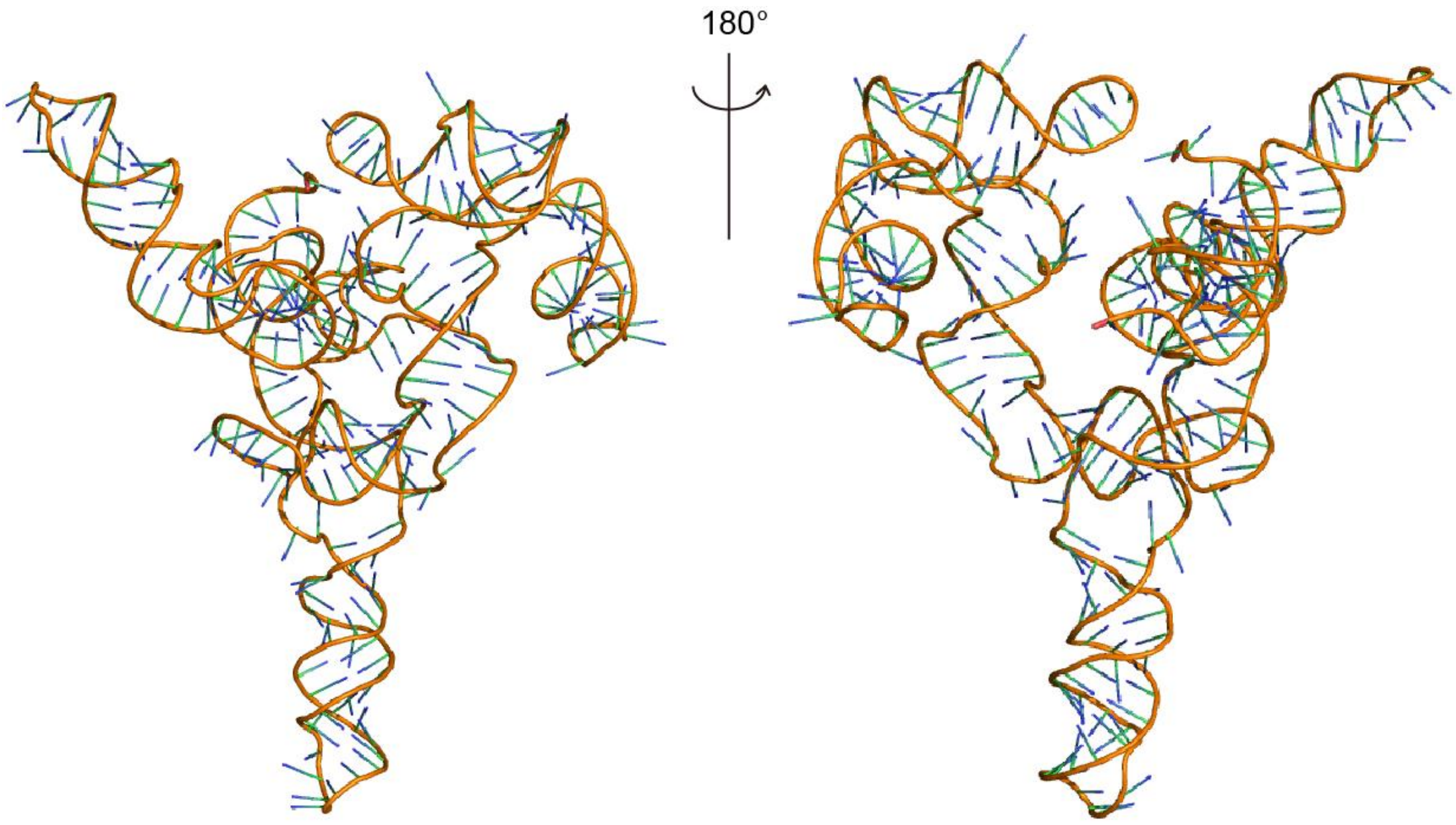

Fig S2. The predicted RNA tertiary structure of LMP2A-IT1 full-length transcript.

The full-length sequence of LMP2A-IT was input into the 3dRNA web server, and the tertiary structure of RNA was generated with the default parameters. The structure of model 1 is shown in the figure.

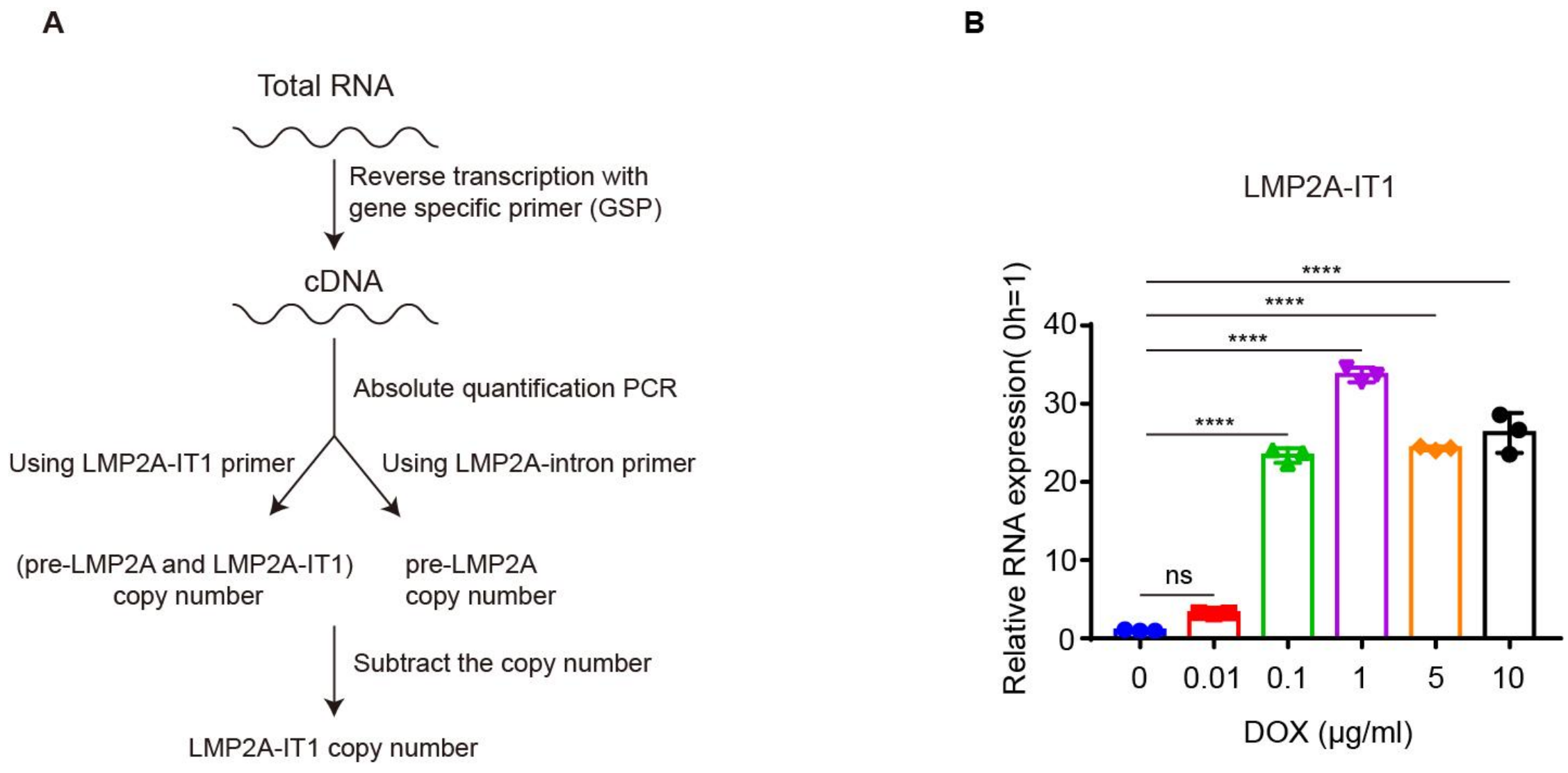

Fig S3. Establishment of gene-specific RT-PCR methods.

(A) A schematic diagram of the specific RT-PCR strategy. Briefly, total RNA was transcribed with gene specific primer targeting LMP2A-IT1, followed by absolute quantification PCR with two sets of primer. The copy number amplified by LMP2A-IT1 primers is subtracted from the copy number of the LMP2A-intron primers to obtain the copy number specific for LMP2A-IT1 transcript.

(B) Effect of Doxycycline on inducing LMP2A-IT1 expression. CNE2-EBV cells harboring the LMP2A-IT1 inducible expression system were treated with DOX at the indicated concentrations for 24 h, followed by RT-qPCR analysis to detect LMP2A-IT1 expression changes. Data are mean ± SD. n=3. One-way analysis of variance (ANOVA) was carried out with Dunnett's test for multiple comparisons. ns, no significance; ****P < 0.0001.

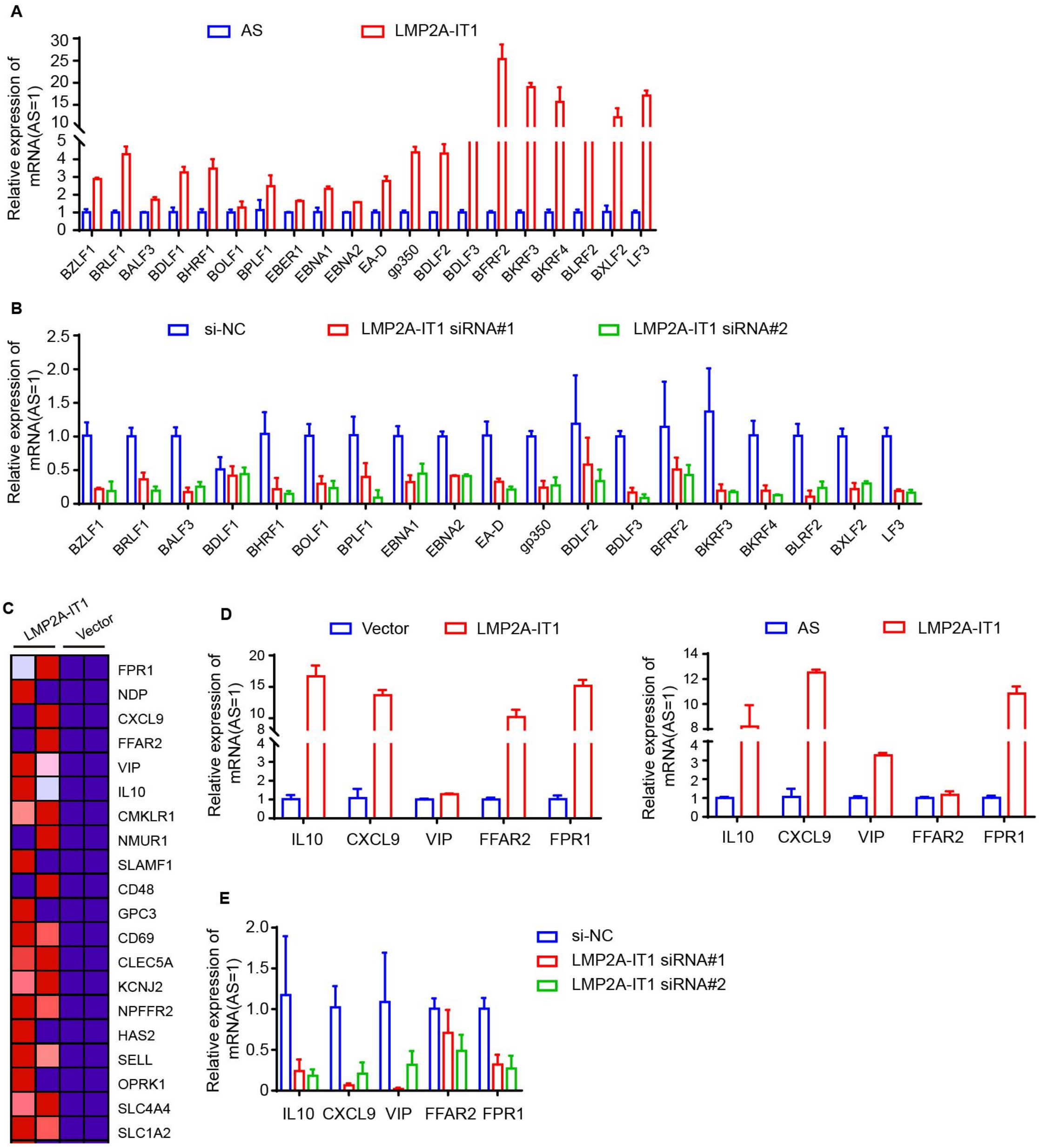

Fig S4. Validation the expression of EBV genes and inflammatory response pathway genes.

(A) Validation the upregulation of EBV genes. CNE2-EBV cells harboring the inducible expression system (LMP2A-IT1 or antisense(AS)) were induced with 1ug/ml Doxycycline, followed by RT-qPCR analysis of the expression changes of the representative EBV genes.

(B) Validation the downregulation of EBV genes. CNE2-EBV cells were transfected with siRNA targeting LMP2A-IT1, followed by RT-qPCR analysis of the expression changes of the representative EBV genes.

(C) Upregulated inflammatory response pathway genes identified in GSEA

analysis.

(D) Validation the upregulation of inflammatory response pathway genes. CNE2-EBV cells harboring the inducible expression system (LMP2A-IT1, vector or antisense(AS)) were induced with 1ug/ml Doxycycline, followed by RT-qPCR analysis of the expression changes of IL10, CXCL9, VIP, FPR1, FFAR2.

(E) Validation the downregulation of inflammatory response pathway genes. CNE2-EBV cells were transfected with siRNA targeting LMP2A-IT1, followed by RT-qPCR analysis of the expression changes of IL10, CXCL9, VIP, FPR1, FFAR2.

The full-length sequence of LMP2A-IT1

CTAGACAGACAGTGTGGCTAAGGGAGTGTGTGCCAGTTAAGGTGATTAGCTAAGGCAT
TCCCAGTAA*ATGGAGGGAGAGTCAGTCAGGCAAGCCTATGACATGGTAATGCCTAGAA*
*GTAAAGAAAGGTTAGTCATAGTAGCTTAG*CTGAACTGGGCCGTGGGGGTCGTCATCAT
CTCCACCGGAACCAGAAGTACCCAAAAGCAGCGTAGGAAGGTGTGGATCACCGGCG
CCCCCGTCTGTCATCGAAGGCGGGCCCCGGTCACCTCCTTTGTTTT

Hypothetical peptide sequence: MEGESVRQAYDMVMPRSKERLVIVA

Fig S5. LMP2A-IT1 has the peptide-coding potential.

ORF finder scans reveal the presence of encodeable open reading frames in the LMP2A-IT1 sequence. The full ORF sequence is underlined, and the bold font is the potential peptide sequence translated from the ORF.

Table S1 part a

		Prot name	prot_hit_num	prot_acc	prot_desc	prot_score	prot_mass	prot_matches	prot_matches_sig	prot_sequences	prot_sequences_sig	prot_cover	prot_pi	prot_tax_str	prot_tax_id	prot_seq	pep_query	pep_rank	pep_isbold	pep_isunique	pep_exp_mz	pep_exp_mr
P08779	K1C16_HUMAN	K1C16	12	sp|P08779|K1C16_HUMAN	Keratin, type I cytoskeletal 16 OS=Homo sapiens OX=9606 GN=KRT16 PE=1 SV=4	656	51578	24	18	18	14	36.6	4.99	Homo sapiens	9606	MTTCSRQFTSS	185	1	1	0	373.7044	745.3942
P04259	K2C6B_HUMAN	K2C6B	23	sp|P04259|K2C6B_HUMAN	Keratin, type II cytoskeletal 6B OS=Homo sapiens OX=9606 GN=KRT6B PE=1 SV=5	497	60315	33	17	23	13	32.4	8.09	Homo sapiens	9606	MASTSTTIRSH	60	1	0	0	324.6735	647.3324
P48668	K2C6C_HUMAN	K2C6C	24	sp|P48668|K2C6C_HUMAN	Keratin, type II cytoskeletal 6C OS=Homo sapiens OX=9606 GN=KRT6C PE=1 SV=3	486	60273	33	17	24	14	34.6	8.09	Homo sapiens	9606	MASTSTTIRSH	60	1	0	0	324.6735	647.3324
Q6S8J3	POTEE_HUMAN	POTEE	25	sp|Q6S8J3|POTEE_HUMAN	POTE ankyrin domain family member E OS=Homo sapiens OX=9606 GN=POTEE PE=2 SV=3	473	122882	30	20	12	8	11.3	5.83	Homo sapiens	9606	MVVEVDSMPAA	38	1	0	0	316.1928	630.3711
P38159	RBMX_HUMAN	RBMX	46	sp|P38159|RBMX_HUMAN	RNA-binding motif protein, X chromosome OS=Homo sapiens OX=9606 GN=RBMX PE=1 SV=3	222	42306	14	9	14	9	32.5	10.06	Homo sapiens	9606	MVEADRPGKLF	143	1	1	1	359.1643	716.3141
Q1KMD3	HNRL2_HUMAN	HNRL2	47	sp|Q1KMD3|HNRL2_HUMAN	Heterogeneous nuclear ribonucleoprotein U-like protein 2 OS=Homo sapiens OX=9606 GN=H	188	85622	11	6	11	6	14.3	4.85	Homo sapiens	9606	MEVKRLKVTEL	45	1	1	1	317.2219	632.4292
Q6PEY2	TBA3E_HUMAN	TBA3E	64	sp|Q6PEY2|TBA3E_HUMAN	Tubulin alpha-3E chain OS=Homo sapiens OX=9606 GN=TUBA3E PE=1 SV=2	100	50568	4	2	4	2	13.1	5	Homo sapiens	9606	MRECISIHVGQ	299	2	0	1	406.2137	810.4128
Q86YZ3	HORN_HUMAN	HORN	83	sp|Q86YZ3|HORN_HUMAN	Hornerin OS=Homo sapiens OX=9606 GN=HRNR PE=1 SV=2	64	283140	2	1	2	1	1.2	10.05	Homo sapiens	9606	MPKLLQGVITV	1474	3	0	1	318.1541	1268.5872
Q9HAV4	XPO5_HUMAN	XPO5	89	sp|Q9HAV4|XPO5_HUMAN	Exportin-5 OS=Homo sapiens OX=9606 GN=XPO5 PE=1 SV=1	57	138332	1	1	1	1	0.8	5.56	Homo sapiens	9606	MAMDQVNALCE	1183	1	1	1	577.3716	1152.7286
Q02878	RL6_HUMAN	RL6	94	sp|Q02878|RL6_HUMAN	60S ribosomal protein L6 OS=Homo sapiens OX=9606 GN=RPL6 PE=1 SV=3	47	32765	1	1	1	1	5.2	10.59	Homo sapiens	9606	MAGEKVEKPDT	2000	1	1	1	763.4728	1524.9311
P09651	ROA1_HUMAN	ROA1	98	sp|P09651|ROA1_HUMAN	Heterogeneous nuclear ribonucleoprotein A1 OS=Homo sapiens OX=9606 GN=HNRNPA1 PE=1 SV	44	38837	3	1	3	1	7.5	9.17	Homo sapiens	9606	MSKSESPKEPE	3	1	1	0	300.708	599.4015
Q32P51	RA1L2_HUMAN	RA1L2	99	sp|Q32P51|RA1L2_HUMAN	Heterogeneous nuclear ribonucleoprotein A1-like 2 OS=Homo sapiens OX=9606 GN=HNRNPA1L	44	34375	3	1	3	1	7.2	9.08	Homo sapiens	9606	MSKSASPKEPE	3	1	0	0	300.708	599.4015
P13639	EF2_HUMAN	EF2	101	sp|P13639|EF2_HUMAN	Elongation factor 2 OS=Homo sapiens OX=9606 GN=EEF2 PE=1 SV=4	43	96246	1	1	1	1	1.5	6.41	Homo sapiens	9606	MVNFTVDQIRA	1851	1	1	1	722.8914	1443.7683
Q7Z4T9	CFA91_HUMAN	CFA91	107	sp|Q7Z4T9|CFA91_HUMAN	Cilia- and flagella-associated protein 91 OS=Homo sapiens OX=9606 GN=MAATS1 PE=1 SV=3	43	90127	3	1	3	1	2.9	8.73	Homo sapiens	9606	MSHAVTIEEPQ	73	1	0	0	329.6984	657.3822
Q12769	NU160_HUMAN	NU160	110	sp|Q12769|NU160_HUMAN	Nuclear pore complex protein Nup160 OS=Homo sapiens OX=9606 GN=NUP160 PE=1 SV=3	43	164355	2	1	2	1	0.8	5.31	Homo sapiens	9606	MLHLSAAPPAP	73	1	0	0	329.6984	657.3822
P35900	K1C20_HUMAN	K1C20	118	sp|P35900|K1C20_HUMAN	Keratin, type I cytoskeletal 20 OS=Homo sapiens OX=9606 GN=KRT20 PE=1 SV=1	40	48514	4	2	2	1	4.7	5.52	Homo sapiens	9606	MDFSRRSFHRS	1077	10	0	1	554.277	1106.5394
Q93034	CUL5_HUMAN	CUL5	123	sp|Q93034|CUL5_HUMAN	Cullin-5 OS=Homo sapiens OX=9606 GN=CUL5 PE=1 SV=4	40	91468	1	1	1	1	0.8	8.08	Homo sapiens	9606	MATSNLLKNKG	102	1	0	1	343.2226	684.4307
Q14103	HNRPD_HUMAN	HNRPD	125	sp|Q14103|HNRPD_HUMAN	Heterogeneous nuclear ribonucleoprotein D0 OS=Homo sapiens OX=9606 GN=HNRNPD PE=1 SV=	40	38581	3	1	3	1	6.2	7.62	Homo sapiens	9606	MSEEQFGGDGA	226	1	1	1	386.2217	770.4289
Q9H3Q3	G3ST2_HUMAN	G3ST2	128	sp|Q9H3Q3|G3ST2_HUMAN	Galactose-3-O-sulfotransferase 2 OS=Homo sapiens OX=9606 GN=GAL3ST2 PE=1 SV=2	39	46366	1	1	1	1	1.5	10.03	Homo sapiens	9606	MMSMLGGLQRY	52	1	0	1	321.6825	641.3505
Q5SNV9	CA167_HUMAN	CA167	129	sp|Q5SNV9|CA167_HUMAN	Uncharacterized protein C1orf167 OS=Homo sapiens OX=9606 GN=C1orf167 PE=2 SV=2	39	164602	3	1	3	1	1.5	10.71	Homo sapiens	9606	MTRMSCPWGSY	136	1	0	1	356.7217	711.4288
Q01780	EXOSX_HUMAN	EXOSX	162	sp|Q01780|EXOSX_HUMAN	Exosome component 10 OS=Homo sapiens OX=9606 GN=EXOSC10 PE=1 SV=2	36	101566	1	1	1	1	0.7	8.68	Homo sapiens	9606	MAPPSTREPRV	102	3	0	1	343.2226	684.4307
A6NI56	CC154_HUMAN	CC154	170	sp|A6NI56|CC154_HUMAN	Coiled-coil domain-containing protein 154 OS=Homo sapiens OX=9606 GN=CCDC154 PE=2 SV=	34	76422	2	1	2	1	3.1	8.62	Homo sapiens	9606	MSELADSGPSG	185	1	0	1	373.7044	745.3942
P06746	DPOLB_HUMAN	DPOLB	171	sp|P06746|DPOLB_HUMAN	DNA polymerase beta OS=Homo sapiens OX=9606 GN=POLB PE=1 SV=3	34	38325	1	1	1	1	1.8	9.01	Homo sapiens	9606	MSKRKAPQETL	185	1	0	1	373.7044	745.3942
Q13459	MYO9B_HUMAN	MYO9B	174	sp|Q13459|MYO9B_HUMAN	Unconventional myosin-IXb OS=Homo sapiens OX=9606 GN=MYO9B PE=1 SV=3	33	244846	4	1	4	1	1.6	8.91	Homo sapiens	9606	MSVKEAGSSGR	53	1	0	1	321.7007	641.3869
A0A1B0G	MRCOL_HUMAN	MRCOL	175	sp|A0A1B0GUY1|MRCOL_HUMA	MARCO-like protein OS=Homo sapiens OX=9606 GN=MARCOL PE=2 SV=1	33	29758	2	1	2	1	5.3	10.29	Homo sapiens	9606	MRAFIFFLFML	53	1	0	0	321.7007	641.3869
Q86W50	MET16_HUMAN	MET16	176	sp|Q86W50|MET16_HUMAN	RNA N6-adenosine-methyltransferase METTL16 OS=Homo sapiens OX=9606 GN=METTL16 PE=1 SV	33	64379	2	1	2	1	2.3	8.08	Homo sapiens	9606	MALSKSMHARN	53	1	0	0	321.7007	641.3869
Q15428	SF3A2_HUMAN	SF3A2	183	sp|Q15428|SF3A2_HUMAN	Splicing factor 3A subunit 2 OS=Homo sapiens OX=9606 GN=SF3A2 PE=1 SV=2	32	49338	1	1	1	1	2.2	9.65	Homo sapiens	9606	MDFQHRPGGKT	870	1	1	1	519.2684	1036.5223
Q8IZY2	ABCA7_HUMAN	ABCA7	188	sp|Q8IZY2|ABCA7_HUMAN	ATP-binding cassette sub-family A member 7 OS=Homo sapiens OX=9606 GN=ABCA7 PE=1 SV=3	32	236254	4	1	4	1	1.1	6.85	Homo sapiens	9606	MAFWTQLMLLL	42	1	1	1	316.6905	631.3665
Q13627	DYR1A_HUMAN	DYR1A	204	sp|Q13627|DYR1A_HUMAN	Dual specificity tyrosine-phosphorylation-regulated kinase 1A OS=Homo sapiens OX=9606	31	86043	2	1	2	1	1.8	8.9	Homo sapiens	9606	MHTGGETSACK	34	1	0	0	315.2039	628.3932
Q9UQ80	PA2G4_HUMAN	PA2G4	207	sp|Q9UQ80|PA2G4_HUMAN	Proliferation-associated protein 2G4 OS=Homo sapiens OX=9606 GN=PA2G4 PE=1 SV=3	31	44101	3	1	3	1	7.6	6.13	Homo sapiens	9606	MSGEDEQQEQT	691	1	1	1	491.2508	980.4871
Q99569	PKP4_HUMAN	PKP4	208	sp|Q99569|PKP4_HUMAN	Plakophilin-4 OS=Homo sapiens OX=9606 GN=PKP4 PE=1 SV=2	31	132585	2	1	2	1	1.8	9.07	Homo sapiens	9606	MPAPEQASLVE	996	3	0	1	539.2711	1076.5276
O60353	FZD6_HUMAN	FZD6	210	sp|O60353|FZD6_HUMAN	Frizzled-6 OS=Homo sapiens OX=9606 GN=FZD6 PE=1 SV=2	31	80951	1	1	1	1	0.8	8.29	Homo sapiens	9606	MEMFTFLLTCI	209	1	0	1	381.1923	760.3701
Q7Z7L7	ZER1_HUMAN	ZER1	219	sp|Q7Z7L7|ZER1_HUMAN	Protein zer-1 homolog OS=Homo sapiens OX=9606 GN=ZER1 PE=1 SV=1	30	89481	1	1	1	1	0.8	5.43	Homo sapiens	9606	MASDTPESLMA	285	1	0	1	402.2119	802.4092
Q96PN7	TREF1_HUMAN	TREF1	221	sp|Q96PN7|TREF1_HUMAN	Transcriptional-regulating factor 1 OS=Homo sapiens OX=9606 GN=TRERF1 PE=1 SV=1	30	132914	2	2	2	2	1.1	6.26	Homo sapiens	9606	MGDQQLYKTNH	33	3	0	1	315.1846	628.3547
Q14008	CKAP5_HUMAN	CKAP5	222	sp|Q14008|CKAP5_HUMAN	Cytoskeleton-associated protein 5 OS=Homo sapiens OX=9606 GN=CKAP5 PE=1 SV=3	30	227062	2	1	2	1	0.6	7.95	Homo sapiens	9606	MGDDSEWLKLP	168	1	0	0	368.716	735.4174
Q9BVN2	RUSC1_HUMAN	RUSC1	224	sp|Q9BVN2|RUSC1_HUMAN	RUN and SH3 domain-containing protein 1 OS=Homo sapiens OX=9606 GN=RUSC1 PE=1 SV=3	30	97354	3	1	3	1	2.8	5.9	Homo sapiens	9606	MLSPQRALLCN	226	2	0	1	386.2217	770.4289
Q9H211	CDT1_HUMAN	CDT1	228	sp|Q9H211|CDT1_HUMAN	DNA replication factor Cdt1 OS=Homo sapiens OX=9606 GN=CDT1 PE=1 SV=3	29	60923	1	1	1	1	1.3	9.82	Homo sapiens	9606	MEQRRVTDFFA	382	1	1	1	424.2271	846.4397
A6NC98	CC88B_HUMAN	CC88B	233	sp|A6NC98|CC88B_HUMAN	Coiled-coil domain-containing protein 88B OS=Homo sapiens OX=9606 GN=CCDC88B PE=1 SV=	28	165166	4	1	2	1	1.2	5.09	Homo sapiens	9606	MEGGKGPRLRD	201	6	0	0	379.7246	757.4346
Q96Q89	KI20B_HUMAN	KI20B	244	sp|Q96Q89|KI20B_HUMAN	Kinesin-like protein KIF20B OS=Homo sapiens OX=9606 GN=KIF20B PE=1 SV=3	28	211868	3	1	3	1	2.2	5.54	Homo sapiens	9606	MESNFNQEGVP	1275	3	0	1	595.8252	1189.6359
Q15633	TRBP2_HUMAN	TRBP2	245	sp|Q15633|TRBP2_HUMAN	RISC-loading complex subunit TARBP2 OS=Homo sapiens OX=9606 GN=TARBP2 PE=1 SV=3	28	39642	1	1	1	1	1.6	6.11	Homo sapiens	9606	MSEEEQGSGTT	168	2	0	1	368.716	735.4174
O94906	PRP6_HUMAN	PRP6	246	sp|O94906|PRP6_HUMAN	Pre-mRNA-processing factor 6 OS=Homo sapiens OX=9606 GN=PRPF6 PE=1 SV=1	28	107656	2	1	2	1	1.4	8.49	Homo sapiens	9606	MNKKKKPFLGM	104	2	0	1	343.713	685.4115
Q96A84	EMID1_HUMAN	EMID1	247	sp|Q96A84|EMID1_HUMAN	EMI domain-containing protein 1 OS=Homo sapiens OX=9606 GN=EMID1 PE=2 SV=1	28	45834	2	1	1	1	2	9.31	Homo sapiens	9606	MGGPRAWALLC	987	3	0	1	358.8733	1073.5981
Q8WWM7	ATX2L_HUMAN	ATX2L	257	sp|Q8WWM7|ATX2L_HUMAN	Ataxin-2-like protein OS=Homo sapiens OX=9606 GN=ATXN2L PE=1 SV=2	27	113589	3	1	3	1	3.2	8.7	Homo sapiens	9606	MLKPQPLQQPS	1023	1	1	1	544.2737	1086.5328
Q8IZP2	ST134_HUMAN	ST134	258	sp|Q8IZP2|ST134_HUMAN	Putative protein FAM10A4 OS=Homo sapiens OX=9606 GN=ST13P4 PE=5 SV=1	26	27561	1	1	1	1	3.8	5.01	Homo sapiens	9606	MDPRKVNELRA	1124	1	1	1	563.8162	1125.6179
Q8IW93	ARHGJ_HUMAN	ARHGJ	261	sp|Q8IW93|ARHGJ_HUMAN	Rho guanine nucleotide exchange factor 19 OS=Homo sapiens OX=9606 GN=ARHGEF19 PE=1 SV	26	89997	3	1	3	1	3.5	7.31	Homo sapiens	9606	MDCGPPATLQP	308	10	1	1	408.2351	814.4557
Q7Z4M0	RE114_HUMAN	RE114	262	sp|Q7Z4M0|RE114_HUMAN	Meiotic recombination protein REC114 OS=Homo sapiens OX=9606 GN=REC114 PE=2 SV=2	26	29650	1	1	1	1	2.3	5.48	Homo sapiens	9606	MAEAGKVPLSL	71	1	0	1	329.2247	656.4349
Q86TB3	ALPK2_HUMAN	ALPK2	264	sp|Q86TB3|ALPK2_HUMAN	Alpha-protein kinase 2 OS=Homo sapiens OX=9606 GN=ALPK2 PE=2 SV=3	26	239887	2	1	2	1	0.7	5.14	Homo sapiens	9606	MKDSEGPQRPP	33	2	0	1	315.1846	628.3547
Q15643	TRIPB_HUMAN	TRIPB	265	sp|Q15643|TRIPB_HUMAN	Thyroid receptor-interacting protein 11 OS=Homo sapiens OX=9606 GN=TRIP11 PE=1 SV=3	26	228131	4	1	4	1	1.5	5.18	Homo sapiens	9606	MSSWLGGLGSG	37	5	0	0	316.1874	630.3602
Q92526	TCPW_HUMAN	TCPW	269	sp|Q92526|TCPW_HUMAN	T-complex protein 1 subunit zeta-2 OS=Homo sapiens OX=9606 GN=CCT6B PE=1 SV=5	26	58355	3	1	2	1	3.8	6.85	Homo sapiens	9606	MAAIKAVNSKA	730	6	0	1	495.7668	989.5191
Q96RW7	HMCN1_HUMAN	HMCN1	271	sp|Q96RW7|HMCN1_HUMAN	Hemicentin-1 OS=Homo sapiens OX=9606 GN=HMCN1 PE=1 SV=2	25	623265	4	2	4	2	0.6	6.07	Homo sapiens	9606	MISWEVVHTVF	102	5	0	1	343.2226	684.4307
O75052	CAPON_HUMAN	CAPON	272	sp|O75052|CAPON_HUMAN	Carboxyl-terminal PDZ ligand of neuronal nitric oxide synthase protein OS=Homo sapien	25	56457	1	1	1	1	1.4	5.89	Homo sapiens	9606	MPSKTKYNLVD	407	1	1	1	428.7665	855.5184
Q9UMN6	KMT2B_HUMAN	KMT2B	275	sp|Q9UMN6|KMT2B_HUMAN	Histone-lysine N-methyltransferase 2B OS=Homo sapiens OX=9606 GN=KMT2B PE=1 SV=1	25	297664	3	1	3	1	1.3	8.59	Homo sapiens	9606	MAAAAGGGSCP	122	5	0	1	350.7345	699.4545
O75526	RMXL2_HUMAN	RMXL2	276	sp|O75526|RMXL2_HUMAN	RNA-binding motif protein, X-linked-like-2 OS=Homo sapiens OX=9606 GN=RBMXL2 PE=1 SV=	25	42959	3	1	3	1	6.1	10.33	Homo sapiens	9606	MVEADRPGKLF	385	2	0	1	424.699	847.3834
Q9Y5A7	NUB1_HUMAN	NUB1	277	sp|Q9Y5A7|NUB1_HUMAN	NEDD8 ultimate buster 1 OS=Homo sapiens OX=9606 GN=NUB1 PE=1 SV=2	24	71235	2	1	2	1	1.8	5.71	Homo sapiens	9606	MAQKKYLQAKL	40	9	0	0	316.6902	631.3658
Q6PD62	CTR9_HUMAN	CTR9	280	sp|Q6PD62|CTR9_HUMAN	RNA polymerase-associated protein CTR9 homolog OS=Homo sapiens OX=9606 GN=CTR9 PE=1 S	24	134332	1	1	1	1	0.5	6.32	Homo sapiens	9606	MSRGSIEIPLR	118	1	1	1	349.2038	696.3931
Q14134	TRI29_HUMAN	TRI29	283	sp|Q14134|TRI29_HUMAN	Tripartite motif-containing protein 29 OS=Homo sapiens OX=9606 GN=TRIM29 PE=1 SV=2	24	66478	1	0	1	0	1.9	6.73	Homo sapiens	9606	MEAADASRSNG	1350	1	1	1	607.8068	1213.5991
O95971	BY55_HUMAN	BY55	284	sp|O95971|BY55_HUMAN	CD160 antigen OS=Homo sapiens OX=9606 GN=CD160 PE=1 SV=1	24	20253	2	1	2	1	22.1	6.93	Homo sapiens	9606	MLLEPGRGCCA	71	5	0	1	329.2247	656.4349
Q8WYP5	ELYS_HUMAN	ELYS	285	sp|Q8WYP5|ELYS_HUMAN	Protein ELYS OS=Homo sapiens OX=9606 GN=AHCTF1 PE=1 SV=3	24	254223	1	1	1	1	0.2	6.19	Homo sapiens	9606	MRDLRAQVTSG	71	5	0	1	329.2247	656.4349
A4D126	ISPD_HUMAN	ISPD	286	sp|A4D126|ISPD_HUMAN	D-ribitol-5-phosphate cytidylyltransferase OS=Homo sapiens OX=9606 GN=ISPD PE=1 SV=2	24	50582	1	1	1	1	1.6	5.86	Homo sapiens	9606	MEAGPPGSARP	335	2	0	1	414.2165	826.4185
Q2QGD7	ZXDC_HUMAN	ZXDC	290	sp|Q2QGD7|ZXDC_HUMAN	Zinc finger protein ZXDC OS=Homo sapiens OX=9606 GN=ZXDC PE=1 SV=2	24	91357	1	1	1	1	0.7	8.48	Homo sapiens	9606	MDLPALLPAPT	56	6	0	1	322.7031	643.3916
Q9Y252	RNF6_HUMAN	RNF6	291	sp|Q9Y252|RNF6_HUMAN	E3 ubiquitin-protein ligase RNF6 OS=Homo sapiens OX=9606 GN=RNF6 PE=1 SV=1	24	78444	3	0	2	0	3.1	9.16	Homo sapiens	9606	MNQSRSRSDGG	616	1	1	1	474.2338	946.4531
Q9P0W8	SPAT7_HUMAN	SPAT7	292	sp|Q9P0W8|SPAT7_HUMAN	Spermatogenesis-associated protein 7 OS=Homo sapiens OX=9606 GN=SPATA7 PE=1 SV=3	24	68190	1	1	1	1	1.2	5.9	Homo sapiens	9606	MDGSRRVRATS	177	1	0	1	372.2243	742.434
Q9Y4D2	DGLA_HUMAN	DGLA	299	sp|Q9Y4D2|DGLA_HUMAN	Sn1-specific diacylglycerol lipase alpha OS=Homo sapiens OX=9606 GN=DAGLA PE=1 SV=3	23	116418	2	1	2	1	1	5.95	Homo sapiens	9606	MPGIVVFRRRW	2	8	0	0	300.1803	598.346
Q5TCS8	KAD9_HUMAN	KAD9	300	sp|Q5TCS8|KAD9_HUMAN	Adenylate kinase 9 OS=Homo sapiens OX=9606 GN=AK9 PE=1 SV=2	23	222698	1	1	1	1	0.3	4.96	Homo sapiens	9606	MTSQEKTEEYP	64	1	0	1	326.1892	650.3639
Q12874	SF3A3_HUMAN	SF3A3	301	sp|Q12874|SF3A3_HUMAN	Splicing factor 3A subunit 3 OS=Homo sapiens OX=9606 GN=SF3A3 PE=1 SV=1	23	59154	1	1	1	1	1	5.27	Homo sapiens	9606	METILEQQRRY	64	1	0	1	326.1892	650.3639
P15812	CD1E_HUMAN	CD1E	302	sp|P15812|CD1E_HUMAN	T-cell surface glycoprotein CD1e, membrane-associated OS=Homo sapiens OX=9606 GN=CD1E	23	44169	1	1	1	1	1.3	8.87	Homo sapiens	9606	MLLLFLLFEGL	64	1	0	1	326.1892	650.3639
P46940	IQGA1_HUMAN	IQGA1	303	sp|P46940|IQGA1_HUMAN	Ras GTPase-activating-like protein IQGAP1 OS=Homo sapiens OX=9606 GN=IQGAP1 PE=1 SV=1	23	189761	3	1	3	1	1.1	6.08	Homo sapiens	9606	MSAADEVDGLG	46	2	0	0	318.1979	634.3812
P06400	RB_HUMAN	RB	304	sp|P06400|RB_HUMAN	Retinoblastoma-associated protein OS=Homo sapiens OX=9606 GN=RB1 PE=1 SV=2	23	106947	2	1	2	1	1.6	8.13	Homo sapiens	9606	MPPKTPRKTAA	46	2	0	0	318.1979	634.3812
Q6ZN44	UNC5A_HUMAN	UNC5A	305	sp|Q6ZN44|UNC5A_HUMAN	Netrin receptor UNC5A OS=Homo sapiens OX=9606 GN=UNC5A PE=1 SV=3	23	94412	2	1	2	1	1.5	6.33	Homo sapiens	9606	MAVRPGLWPAL	46	2	0	1	318.1979	634.3812
Q6ZR62	RTL4_HUMAN	RTL4	308	sp|Q6ZR62|RTL4_HUMAN	Retrotransposon Gag-like protein 4 OS=Homo sapiens OX=9606 GN=RTL4 PE=2 SV=2	23	35119	1	1	1	1	2.3	5.5	Homo sapiens	9606	MEKCTKSSSTM	302	2	0	1	406.7524	811.4903
P40429	RL13A_HUMAN	RL13A	312	sp|P40429|RL13A_HUMAN	60S ribosomal protein L13a OS=Homo sapiens OX=9606 GN=RPL13A PE=1 SV=2	23	23619	2	1	2	1	6.4	10.94	Homo sapiens	9606	MAEVQVLVLDG	102	6	0	1	343.2226	684.4307
Q96Q35	AL2SB_HUMAN	AL2SB	315	sp|Q96Q35|AL2SB_HUMAN	Amyotrophic lateral sclerosis 2 chromosomal region candidate gene 12 protein OS=Homo	23	52748	2	1	2	1	3.8	6.29	Homo sapiens	9606	MYPNPLIYCTC	19	1	0	1	311.1672	620.3199
Q13488	VPP3_HUMAN	VPP3	318	sp|Q13488|VPP3_HUMAN	V-type proton ATPase 116 kDa subunit a isoform 3 OS=Homo sapiens OX=9606 GN=TCIRG1 PE	22	93650	2	1	2	1	1.6	6.66	Homo sapiens	9606	MGSMFRSEEVA	102	7	0	1	343.2226	684.4307
Q96NI6	LRFN5_HUMAN	LRFN5	328	sp|Q96NI6|LRFN5_HUMAN	Leucine-rich repeat and fibronectin type-III domain-containing protein 5 OS=Homo sapi	22	80421	1	1	1	1	0.8	7.27	Homo sapiens	9606	MEKILFYLFLI	89	1	0	1	335.6985	669.3824
Q9H6T0	ESRP2_HUMAN	ESRP2	329	sp|Q9H6T0|ESRP2_HUMAN	Epithelial splicing regulatory protein 2 OS=Homo sapiens OX=9606 GN=ESRP2 PE=1 SV=1	22	79206	1	1	1	1	1	6.27	Homo sapiens	9606	MTPPPPPPPPP	121	2	0	1	350.709	699.4034
Q9NYI0	PSD3_HUMAN	PSD3	330	sp|Q9NYI0|PSD3_HUMAN	PH and SEC7 domain-containing protein 3 OS=Homo sapiens OX=9606 GN=PSD3 PE=1 SV=2	22	116646	3	1	3	1	1.8	5.68	Homo sapiens	9606	MEGRSAAAETF	68	5	0	0	328.1738	654.3331
P35711	SOX5_HUMAN	SOX5	331	sp|P35711|SOX5_HUMAN	Transcription factor SOX-5 OS=Homo sapiens OX=9606 GN=SOX5 PE=1 SV=3	22	84201	2	1	2	1	1.6	6.15	Homo sapiens	9606	MLTDPDLPQEF	68	5	0	0	328.1738	654.3331
A8MTL0	IQCF5_HUMAN	IQCF5	332	sp|A8MTL0|IQCF5_HUMAN	IQ domain-containing protein F5 OS=Homo sapiens OX=9606 GN=IQCF5 PE=2 SV=2	22	18379	1	1	1	1	4.1	10.05	Homo sapiens	9606	MGPEEKTIMTE	102	8	0	1	343.2226	684.4307
Q9Y666	S12A7_HUMAN	S12A7	333	sp|Q9Y666|S12A7_HUMAN	Solute carrier family 12 member 7 OS=Homo sapiens OX=9606 GN=SLC12A7 PE=1 SV=3	21	120283	3	1	3	1	2.2	6.28	Homo sapiens	9606	MPTNFTVVPVE	125	9	1	1	351.6837	701.3528
O75676	KS6A4_HUMAN	KS6A4	334	sp|O75676|KS6A4_HUMAN	Ribosomal protein S6 kinase alpha-4 OS=Homo sapiens OX=9606 GN=RPS6KA4 PE=1 SV=1	21	86122	5	1	4	1	5.4	8.51	Homo sapiens	9606	MGDEDDDESCA	43	2	0	1	317.2087	632.4029
Q8IYW2	CFA46_HUMAN	CFA46	335	sp|Q8IYW2|CFA46_HUMAN	Cilia- and flagella-associated protein 46 OS=Homo sapiens OX=9606 GN=CFAP46 PE=2 SV=3	20	306786	2	1	2	1	0.5	7.07	Homo sapiens	9606	MDLVITQELAR	45	1	0	1	317.2219	632.4292
Q9BZL6	KPCD2_HUMAN	KPCD2	336	sp|Q9BZL6|KPCD2_HUMAN	Serine/threonine-protein kinase D2 OS=Homo sapiens OX=9606 GN=PRKD2 PE=1 SV=3	20	97972	2	1	2	1	1.6	6.39	Homo sapiens	9606	MATAPSYPAGL	45	1	0	1	317.2219	632.4292
Q9H239	MMP28_HUMAN	MMP28	337	sp|Q9H239|MMP28_HUMAN	Matrix metalloproteinase-28 OS=Homo sapiens OX=9606 GN=MMP28 PE=2 SV=2	20	59187	1	1	1	1	1	9.7	Homo sapiens	9606	MVARVGLLLRA	45	1	0	1	317.2219	632.4292
P49788	TIG1_HUMAN	TIG1	338	sp|P49788|TIG1_HUMAN	Retinoic acid receptor responder protein 1 OS=Homo sapiens OX=9606 GN=RARRES1 PE=1 SV	20	33549	1	1	1	1	2.4	8.74	Homo sapiens	9606	MQPRRQRLPAP	281	2	0	1	400.2435	798.4724
Q9NX05	F120C_HUMAN	F120C	348	sp|Q9NX05|F120C_HUMAN	Constitutive coactivator of PPAR-gamma-like protein 2 OS=Homo sapiens OX=9606 GN=FAM1	18	121652	5	0	4	0	2.9	9.19	Homo sapiens	9606	MGVQGFQEFLE	23	1	0	0	311.682	621.3495
Q9NRL3	STRN4_HUMAN	STRN4	351	sp|Q9NRL3|STRN4_HUMAN	Striatin-4 OS=Homo sapiens OX=9606 GN=STRN4 PE=1 SV=2	17	81287	1	0	1	0	2.9	5.21	Homo sapiens	9606	MMEERAAAAVA	3079	1	1	1	1335.6672	2669.3199
Q2M296	MTHSD_HUMAN	MTHSD	352	sp|Q2M296|MTHSD_HUMAN	Methenyltetrahydrofolate synthase domain-containing protein OS=Homo sapiens OX=9606 G	16	42488	1	1	1	1	1.3	8.93	Homo sapiens	9606	MEPRAVGVSKQ	14	5	0	1	306.2165	610.4185
Q5VU92	DC121_HUMAN	DC121	353	sp|Q5VU92|DC121_HUMAN	DDB1- and CUL4-associated factor 12-like protein 1 OS=Homo sapiens OX=9606 GN=DCAF12L	16	51739	1	1	1	1	1.1	8.84	Homo sapiens	9606	MAQQQTGSRKR	14	5	0	1	306.2165	610.4185
Q9NX76	CKLF6_HUMAN	CKLF6	354	sp|Q9NX76|CKLF6_HUMAN	CKLF-like MARVEL transmembrane domain-containing protein 6 OS=Homo sapiens OX=9606 GN	16	20748	1	1	1	1	2.7	5.23	Homo sapiens	9606	MENGAVYSPTT	14	5	0	1	306.2165	610.4185
Q14165	MLEC_HUMAN	MLEC	355	sp|Q14165|MLEC_HUMAN	Malectin OS=Homo sapiens OX=9606 GN=MLEC PE=1 SV=1	16	32385	1	1	1	1	1.7	5.27	Homo sapiens	9606	MLGAWAVEGTA	14	5	0	1	306.2165	610.4185
Q8TD57	DYH3_HUMAN	DYH3	356	sp|Q8TD57|DYH3_HUMAN	Dynein heavy chain 3, axonemal OS=Homo sapiens OX=9606 GN=DNAH3 PE=2 SV=1	16	473776	3	1	3	1	0.5	6.04	Homo sapiens	9606	MGATGRLELTL	290	8	0	1	404.2034	806.3922
Q92616	GCN1_HUMAN	GCN1	357	sp|Q92616|GCN1_HUMAN	eIF-2-alpha kinase activator GCN1 OS=Homo sapiens OX=9606 GN=GCN1 PE=1 SV=6	15	294967	3	1	3	1	1.2	7.29	Homo sapiens	9606	MAADTQVSETL	4	1	1	1	303.1778	604.341

Table S1 part b

		pep_exp_z	pep_calc_mr	pep_delta	pep_miss	pep_score	pep_expect	pep_res_befo	pep_seq	pep_res_after	pep_var_mod	pep_var_mod_pos	pep_scan_title		
P08779	K1C16_HUMAN	2	745.397	-0.0028	0	34.4	0.01	K	TIEDLR	N			5318-2-IT1-135KD.1940.1940.2 File:~5318-2-IT1-135KD.raw~, NativeID:~controllerType=0 controllerNumber=1 scan=1940~		
P04259	K2C6B_HUMAN	2	647.3312	0.0012	0	23.96	0.078	R	GEMALK	D			5318-2-IT1-135KD.1597.1597.2 File:~5318-2-IT1-135KD.raw~, NativeID:~controllerType=0 controllerNumber=1 scan=1597~		
P48668	K2C6C_HUMAN	2	647.3312	0.0012	0	23.96	0.078	R	GEMALK	D			5318-2-IT1-135KD.1597.1597.2 File:~5318-2-IT1-135KD.raw~, NativeID:~controllerType=0 controllerNumber=1 scan=1597~		
Q6S8J3	POTEE_HUMAN	2	630.3701	0.001	0	19.58	0.37	K	ILTER	G			5318-2-IT1-135KD.1528.1528.2 File:~5318-2-IT1-135KD.raw~, NativeID:~controllerType=0 controllerNumber=1 scan=1528~		
P38159	RBMX_HUMAN	2	716.3129	0.0012	0	18.69	0.065	R	DYTYR	D			5318-2-IT1-135KD.1727.1727.2 File:~5318-2-IT1-135KD.raw~, NativeID:~controllerType=0 controllerNumber=1 scan=1727~		
Q1KMD3	HNRL2_HUMAN	2	632.4261	0.0031	0	20.26	0.01	K	LLLFK	T			5318-2-IT1-135KD.3270.3270.2 File:~5318-2-IT1-135KD.raw~, NativeID:~controllerType=0 controllerNumber=1 scan=3270~		
Q6PEY2	TBA3E_HUMAN	2	810.4123	0.0004	0	3.3	4.3	R	LSVDYSK	K			5318-2-IT1-135KD.1870.1870.2 File:~5318-2-IT1-135KD.raw~, NativeID:~controllerType=0 controllerNumber=1 scan=1870~		
Q86YZ3	HORN_HUMAN	4	1268.597	-0.0098	1	0.6	14	R	QGSGSGHSPSRGR	H			5318-2-IT1-135KD.1035.1035.4 File:~5318-2-IT1-135KD.raw~, NativeID:~controllerType=0 controllerNumber=1 scan=1035~		
Q9HAV4	XPO5_HUMAN	2	1152.723	0.0056	0	57.46	1.90E-06	K	LLDNLLALIR	T			5318-2-IT1-135KD.4572.457	emPAI	0.02
Q02878	RL6_HUMAN	2	1524.9239	0.0072	0	47.48	2.30E-05	K	ASITPGTILIILTGR	H			5318-2-IT1-135KD.4613.461	emPAI	0.1
P09651	ROA1_HUMAN	2	599.4006	0.0008	0	15.18	0.57	K	IVIQK	Y			5318-2-IT1-135KD.1522.1522.2 File:~5318-2-IT1-135KD.raw~, NativeID:~controllerType=0 controllerNumber=1 scan=1522~		
Q32P51	RA1L2_HUMAN	2	599.4006	0.0008	0	15.18	0.57	K	IVIQK	Y			5318-2-IT1-135KD.1522.1522.2 File:~5318-2-IT1-135KD.raw~, NativeID:~controllerType=0 controllerNumber=1 scan=1522~		
P13639	EF2_HUMAN	2	1443.7609	0.0074	1	42.98	0.0013	K	EGIPALDNFLDKL	-			5318-2-IT1-135KD.4623.462	emPAI	0.03
Q7Z4T9	CFA91_HUMAN	2	657.381	0.0012	0	42.68	0.0013	K	LQEIR	L			5318-2-IT1-135KD.1554.1554.2 File:~5318-2-IT1-135KD.raw~, NativeID:~controllerType=0 controllerNumber=1 scan=1554~		
Q12769	NU160_HUMAN	2	657.381	0.0012	0	42.68	0.0013	K	LQEIR	N			5318-2-IT1-135KD.1554.1554.2 File:~5318-2-IT1-135KD.raw~, NativeID:~controllerType=0 controllerNumber=1 scan=1554~		
P35900	K1C20_HUMAN	2	1106.543	-0.0036	1	6.16	4.7	R	QKYEVMAQK	N	Gln->pyro-Glu (N-term	3.000000000.0	5318-2-IT1-135KD.2864.2864.2 File:~5318-2-IT1-135KD.raw~, NativeID:~controllerType=0 controllerNumber=1 scan=2864~		
Q93034	CUL5_HUMAN	2	684.4282	0.0025	0	40	0.00046	K	INLIGR	L			5318-2-IT1-135KD.2763.276	emPAI	0.04
Q14103	HNRPD_HUMAN	2	770.4286	0.0003	0	40.56	0.00089	K	LDPITGR	S			5318-2-IT1-135KD.2183.2183.2 File:~5318-2-IT1-135KD.raw~, NativeID:~controllerType=0 controllerNumber=1 scan=2183~		
Q9H3Q3	G3ST2_HUMAN	2	641.3497	0.0009	0	39.31	0.0021	R	AELGPR	R			5318-2-IT1-135KD.1489.148	emPAI	0.07
Q5SNV9	CA167_HUMAN	2	711.4279	0.0009	0	39.08	0.00083	R	LGLPGAGK	T			5318-2-IT1-135KD.2127.2127.2 File:~5318-2-IT1-135KD.raw~, NativeID:~controllerType=0 controllerNumber=1 scan=2127~		
Q01780	EXOSX_HUMAN	2	684.4282	0.0025	0	35.96	0.0012	R	LLNLGR	H			5318-2-IT1-135KD.2763.276	emPAI	0.03
A6NI56	CC154_HUMAN	2	745.397	-0.0028	0	34.4	0.01	K	TIQNLR	F	2 Deamidated (NQ)	0.002200.0	5318-2-IT1-135KD.1940.1940.2 File:~5318-2-IT1-135KD.raw~, NativeID:~controllerType=0 controllerNumber=1 scan=1940~		
P06746	DPOLB_HUMAN	2	745.397	-0.0028	0	34.4	0.01	K	TLEDLR	K			5318-2-IT1-135KD.1940.194	emPAI	0.09
Q13459	MYO9B_HUMAN	2	641.386	0.0009	0	33.23	0.0047	R	LQKPR	A	Deamidated (NQ)	0.02000.0	5318-2-IT1-135KD.1346.1346.2 File:~5318-2-IT1-135KD.raw~, NativeID:~controllerType=0 controllerNumber=1 scan=1346~		
A0A1B0G	MRCOL_HUMAN	2	641.386	0.0009	0	33.23	0.0047	K	LEKPR	H			5318-2-IT1-135KD.1346.1346.2 File:~5318-2-IT1-135KD.raw~, NativeID:~controllerType=0 controllerNumber=1 scan=1346~		
Q86W50	MET16_HUMAN	2	641.386	0.0009	0	33.23	0.0047	K	LEKPR	R			5318-2-IT1-135KD.1346.1346.2 File:~5318-2-IT1-135KD.raw~, NativeID:~controllerType=0 controllerNumber=1 scan=1346~		
Q15428	SF3A2_HUMAN	2	1036.5189	0.0034	0	32.03	0.012	K	EAPAQPAPEK	V			5318-2-IT1-135KD.1266.126	emPAI	0.07
Q8IZY2	ABCA7_HUMAN	2	631.3653	0.0013	1	22.34	0.21	R	TNKIR	D	Deamidated (NQ)	0.02000.0	5318-2-IT1-135KD.1179.1179.2 File:~5318-2-IT1-135KD.raw~, NativeID:~controllerType=0 controllerNumber=1 scan=1179~		
Q13627	DYR1A_HUMAN	2	628.3908	0.0024	0	31.23	0.016	K	DLILR	M			5318-2-IT1-135KD.2678.2678.2 File:~5318-2-IT1-135KD.raw~, NativeID:~controllerType=0 controllerNumber=1 scan=2678~		
Q9UQ80	PA2G4_HUMAN	2	980.4815	0.0057	0	7.4	3.3	K	SDQDYILK	E			5318-2-IT1-135KD.2437.2437.2 File:~5318-2-IT1-135KD.raw~, NativeID:~controllerType=0 controllerNumber=1 scan=2437~		
Q99569	PKP4_HUMAN	2	1076.5251	0.0026	0	8.15	3.7	K	EQELQFQR	L			5318-2-IT1-135KD.1458.1458.2 File:~5318-2-IT1-135KD.raw~, NativeID:~controllerType=0 controllerNumber=1 scan=1458~		
O60353	FZD6_HUMAN	2	760.3715	-0.0014	0	31.12	0.019	K	TEQVQR	D	Deamidated (NQ)	0.002000.0	5318-2-IT1-135KD.1095.109	emPAI	0.04
Q7Z7L7	ZER1_HUMAN	2	802.4119	-0.0028	0	30.45	0.014	R	CNQLLR	A			5318-2-IT1-135KD.1368.136	emPAI	0.04
Q96PN7	TREF1_HUMAN	2	628.3544	0.0003	0	24.95	0.024	K	GAIPSGK	Q			5318-2-IT1-135KD.1237.1237.2 File:~5318-2-IT1-135KD.raw~, NativeID:~controllerType=0 controllerNumber=1 scan=1237~		
Q14008	CKAP5_HUMAN	2	735.4167	0.0008	0	30.2	0.0074	K	ALEYLK	L			5318-2-IT1-135KD.2330.2330.2 File:~5318-2-IT1-135KD.raw~, NativeID:~controllerType=0 controllerNumber=1 scan=2330~		
Q9BVN2	RUSC1_HUMAN	2	770.4326	-0.0037	0	29.53	0.011	K	INPIWK	I	Deamidated (NQ)	0.020000.0	5318-2-IT1-135KD.2183.2183.2 File:~5318-2-IT1-135KD.raw~, NativeID:~controllerType=0 controllerNumber=1 scan=2183~		
Q9H211	CDT1_HUMAN	2	846.4382	0.0015	0	28.55	0.033	K	QLAQMTR	C			5318-2-IT1-135KD.1619.161	emPAI	0.05
A6NC98	CC88B_HUMAN	2	757.4334	0.0012	0	19.2	0.26	R	LIEVER	S			5318-2-IT1-135KD.2139.2139.2 File:~5318-2-IT1-135KD.raw~, NativeID:~controllerType=0 controllerNumber=1 scan=2139~		
Q96Q89	KI20B_HUMAN	2	1189.6302	0.0057	1	6.25	4.6	K	LKEELSASSAR	T			5318-2-IT1-135KD.1919.1919.2 File:~5318-2-IT1-135KD.raw~, NativeID:~controllerType=0 controllerNumber=1 scan=1919~		
Q15633	TRBP2_HUMAN	2	735.4167	0.0008	0	28.08	0.012	R	ALQYLK	I	Deamidated (NQ)	0.002000.0	5318-2-IT1-135KD.2330.233	emPAI	0.08
O94906	PRP6_HUMAN	2	685.4123	-0.0008	0	27.81	0.023	K	IGDILR	L			5318-2-IT1-135KD.2040.2040.2 File:~5318-2-IT1-135KD.raw~, NativeID:~controllerType=0 controllerNumber=1 scan=2040~		
Q96A84	EMID1_HUMAN	3	1073.608	-0.0099	1	15	0.6	K	VSELTERLK	V			5318-2-IT1-135KD.1872.1872.3 File:~5318-2-IT1-135KD.raw~, NativeID:~controllerType=0 controllerNumber=1 scan=1872~		
Q8WWM7	ATX2L_HUMAN	2	1086.5306	0.0023	0	26.93	0.036	K	STSTPTSPGPR	T			5318-2-IT1-135KD.1329.1329.2 File:~5318-2-IT1-135KD.raw~, NativeID:~controllerType=0 controllerNumber=1 scan=1329~		
Q8IZP2	ST134_HUMAN	2	1125.6254	-0.0075	2	26.5	0.031	M	DPRKVNELR	A			5318-2-IT1-135KD.2419.241	emPAI	0.12
Q8IW93	ARHGJ_HUMAN	2	814.4548	0.0009	0	12.15	1	R	LEADVLR	F			5318-2-IT1-135KD.2079.2079.2 File:~5318-2-IT1-135KD.raw~, NativeID:~controllerType=0 controllerNumber=1 scan=2079~		
Q7Z4M0	RE114_HUMAN	2	656.4333	0.0016	1	26.38	0.022	K	KLAGLR	N			5318-2-IT1-135KD.1439.143	emPAI	0.11
Q86TB3	ALPK2_HUMAN	2	628.3544	0.0003	0	26.2	0.018	K	QPSIGK	S			5318-2-IT1-135KD.1237.1237.2 File:~5318-2-IT1-135KD.raw~, NativeID:~controllerType=0 controllerNumber=1 scan=1237~		
Q15643	TRIPB_HUMAN	2	630.3588	0.0014	0	21.22	0.19	R	ELEIK	L			5318-2-IT1-135KD.1979.1979.2 File:~5318-2-IT1-135KD.raw~, NativeID:~controllerType=0 controllerNumber=1 scan=1979~		
Q92526	TCPW_HUMAN	2	989.5216	-0.0024	0	0.5	20	K	MLVSGAGDIK	L			5318-2-IT1-135KD.2187.2187.2 File:~5318-2-IT1-135KD.raw~, NativeID:~controllerType=0 controllerNumber=1 scan=2187~		
Q96RW7	HMCN1_HUMAN	2	684.4282	0.0025	0	23.1	0.023	K	ILLNGR	K			5318-2-IT1-135KD.2763.2763.2 File:~5318-2-IT1-135KD.raw~, NativeID:~controllerType=0 controllerNumber=1 scan=2763~		
O75052	CAPON_HUMAN	2	855.5178	0.0006	0	25.35	0.022	R	LLNVLQR	Q	Deamidated (NQ)	0.0020000.0	5318-2-IT1-135KD.2265.226	emPAI	0.06
Q9UMN6	KMT2B_HUMAN	2	699.453	0.0015	0	24.75	0.033	K	IILVNK	L	Deamidated (NQ)	0.000020.0	5318-2-IT1-135KD.2654.2654.2 File:~5318-2-IT1-135KD.raw~, NativeID:~controllerType=0 controllerNumber=1 scan=2654~		
O75526	RMXL2_HUMAN	2	847.3824	0.0009	0	11.96	0.62	R	DGYSGPPR	R			5318-2-IT1-135KD.1540.1540.2 File:~5318-2-IT1-135KD.raw~, NativeID:~controllerType=0 controllerNumber=1 scan=1540~		
Q9Y5A7	NUB1_HUMAN	2	631.3653	0.0005	1	15.01	1	R	ELRSK	I			5318-2-IT1-135KD.830.830.2 File:~5318-2-IT1-135KD.raw~, NativeID:~controllerType=0 controllerNumber=1 scan=830~		
Q6PD62	CTR9_HUMAN	2	696.3918	0.0013	0	24.37	0.025	K	APKPER	L			5318-2-IT1-135KD.1436.143	emPAI	0.02
Q14134	TRI29_HUMAN	2	1213.5939	0.0052	0	24.3	0.068	K	NHSTVTVEEAK	A			5318-2-IT1-135KD.1271.127	emPAI	0.05
O95971	BY55_HUMAN	2	656.4333	0.0016	1	24.29	0.035	K	QLRLK	R			5318-2-IT1-135KD.1439.1439.2 File:~5318-2-IT1-135KD.raw~, NativeID:~controllerType=0 controllerNumber=1 scan=1439~		
Q8WYP5	ELYS_HUMAN	2	656.4333	0.0016	1	24.29	0.035	K	QLRIK	H			5318-2-IT1-135KD.1439.143	emPAI	0.01
A4D126	ISPD_HUMAN	2	826.4185	0	0	24.25	0.052	K	SELNHVK	V	Deamidated (NQ)	0.0002000.0	5318-2-IT1-135KD.1235.123	emPAI	0.07
Q2QGD7	ZXDC_HUMAN	2	643.3904	0.0011	0	24.17	0.049	K	AIQLAK	E	Deamidated (NQ)	0.002000.0	5318-2-IT1-135KD.1736.173	emPAI	0.03
Q9Y252	RNF6_HUMAN	2	946.4468	0.0063	1	4.78	7.8	R	DRDSIANR	T	Deamidated (NQ)	0.00000020.0	5318-2-IT1-135KD.2261.2261.2 File:~5318-2-IT1-135KD.raw~, NativeID:~controllerType=0 controllerNumber=1 scan=2261~		
Q9P0W8	SPAT7_HUMAN	2	742.4337	0.0003	0	24.08	0.059	R	ATSVLPR	Y			5318-2-IT1-135KD.2266.226	emPAI	0.05
Q9Y4D2	DGLA_HUMAN	2	598.3439	0.0022	0	9.55	1.2	K	DLVPR	I			5318-2-IT1-135KD.1402.1402.2 File:~5318-2-IT1-135KD.raw~, NativeID:~controllerType=0 controllerNumber=1 scan=1402~		
Q5TCS8	KAD9_HUMAN	2	650.3639	0	0	23.36	0.029	K	YLNLK	R	Deamidated (NQ)	0.00200.0	5318-2-IT1-135KD.2038.203	emPAI	0.02
Q12874	SF3A3_HUMAN	2	650.3639	0	0	23.36	0.029	K	YINLK	A	Deamidated (NQ)	0.00200.0	5318-2-IT1-135KD.2038.203	emPAI	0.06
P15812	CD1E_HUMAN	2	650.3639	0	0	23.36	0.029	R	YLDIK	E			5318-2-IT1-135KD.2038.203	emPAI	0.08
P46940	IQGA1_HUMAN	2	634.3802	0.0009	0	23.3	0.028	R	LAYLR	S			5318-2-IT1-135KD.2209.2209.2 File:~5318-2-IT1-135KD.raw~, NativeID:~controllerType=0 controllerNumber=1 scan=2209~		
P06400	RB_HUMAN	2	634.3802	0.0009	0	23.3	0.028	R	LAYLR	L			5318-2-IT1-135KD.2209.2209.2 File:~5318-2-IT1-135KD.raw~, NativeID:~controllerType=0 controllerNumber=1 scan=2209~		
Q6ZN44	UNC5A_HUMAN	2	634.3802	0.0009	0	23.3	0.028	R	IAYLR	K			5318-2-IT1-135KD.2209.2209.2 File:~5318-2-IT1-135KD.raw~, NativeID:~controllerType=0 controllerNumber=1 scan=2209~		
Q6ZR62	RTL4_HUMAN	2	811.4916	-0.0013	1	23.04	0.027	K	KGPIQLR	E	Deamidated (NQ)	0.0000200.0	5318-2-IT1-135KD.1084.108	emPAI	0.1
P40429	RL13A_HUMAN	2	684.4283	0.0024	0	22.72	0.025	K	QVLLGR	K			5318-2-IT1-135KD.2763.2763.2 File:~5318-2-IT1-135KD.raw~, NativeID:~controllerType=0 controllerNumber=1 scan=2763~		
Q96Q35	AL2SB_HUMAN	2	620.3203	-0.0004	0	22.51	0.047	K	MLQTK	F	Deamidated (NQ)	0.00200.0	5318-2-IT1-135KD.1120.1120.2 File:~5318-2-IT1-135KD.raw~, NativeID:~controllerType=0 controllerNumber=1 scan=1120~		
Q13488	VPP3_HUMAN	2	684.4283	0.0024	0	22.26	0.028	R	IGLGLGR	E			5318-2-IT1-135KD.2763.2763.2 File:~5318-2-IT1-135KD.raw~, NativeID:~controllerType=0 controllerNumber=1 scan=2763~		
Q96NI6	LRFN5_HUMAN	2	669.381	0.0015	0	21.94	0.049	R	NIPGIR	M	Deamidated (NQ)	0.200000.0	5318-2-IT1-135KD.3050.305	emPAI	0.04
Q9H6T0	ESRP2_HUMAN	2	699.4027	0.0007	0	21.88	0.035	R	ALAAAQR	C			5318-2-IT1-135KD.1352.135	emPAI	0.04
Q9NYI0	PSD3_HUMAN	2	654.3337	-0.0006	0	21.69	0.041	K	QHLEK	T	Deamidated (NQ)	0.20000.0	5318-2-IT1-135KD.1074.1074.2 File:~5318-2-IT1-135KD.raw~, NativeID:~controllerType=0 controllerNumber=1 scan=1074~		
P35711	SOX5_HUMAN	2	654.3337	-0.0006	0	21.69	0.041	K	QHLEK	Y	Deamidated (NQ)	0.20000.0	5318-2-IT1-135KD.1074.1074.2 File:~5318-2-IT1-135KD.raw~, NativeID:~controllerType=0 controllerNumber=1 scan=1074~		
A8MTL0	IQCF5_HUMAN	2	684.4282	0.0025	0	21.56	0.032	R	LLNAVR	I			5318-2-IT1-135KD.2763.276	emPAI	0.21
Q9Y666	S12A7_HUMAN	2	701.353	-0.0003	0	0.94	9.8	R	MHTAVK	L	Oxidation (M)	0.500000.0	5318-2-IT1-135KD.1247.1247.2 File:~5318-2-IT1-135KD.raw~, NativeID:~controllerType=0 controllerNumber=1 scan=1247~		
O75676	KS6A4_HUMAN	2	632.401	0.0019	0	21.09	0.018	K	VFLVR	K			5318-2-IT1-135KD.2511.2511.2 File:~5318-2-IT1-135KD.raw~, NativeID:~controllerType=0 controllerNumber=1 scan=2511~		
Q8IYW2	CFA46_HUMAN	2	632.4261	0.0031	0	20.26	0.01	R	LLIFK	H			5318-2-IT1-135KD.3270.3270.2 File:~5318-2-IT1-135KD.raw~, NativeID:~controllerType=0 controllerNumber=1 scan=3270~		
Q9BZL6	KPCD2_HUMAN	2	632.4261	0.0031	0	20.26	0.01	K	ILLFK	H			5318-2-IT1-135KD.3270.3270.2 File:~5318-2-IT1-135KD.raw~, NativeID:~controllerType=0 controllerNumber=1 scan=3270~		
Q9H239	MMP28_HUMAN	2	632.4261	0.0031	0	20.26	0.01	R	LILFK	G			5318-2-IT1-135KD.3270.327	emPAI	0.06
P49788	TIG1_HUMAN	2	798.4712	0.0012	0	20.23	0.04	R	LLQQAAR	A			5318-2-IT1-135KD.1698.169	emPAI	0.1
Q9NX05	F120C_HUMAN	2	621.3486	0.0009	0	18.79	0.17	K	LFQSK	L			5318-2-IT1-135KD.1510.1510.2 File:~5318-2-IT1-135KD.raw~, NativeID:~controllerType=0 controllerNumber=1 scan=1510~		
Q9NRL3	STRN4_HUMAN	2	2669.3371	-0.0171	2	17.4	0.61	R	QLLRQYLEEVGYTDTILD	R	Gln->pyro-Glu (N-term	3.000000000000000	5318-2-IT1-135KD.4743.474	emPAI	0.04
Q2M296	MTHSD_HUMAN	2	610.4166	0.0019	0	16.46	0.055	K	IPILR	S			5318-2-IT1-135KD.2369.236	emPAI	0.08
Q5VU92	DC121_HUMAN	2	610.4166	0.0019	0	16.46	0.055	R	IPLLR	D			5318-2-IT1-135KD.2369.236	emPAI	0.06
Q9NX76	CKLF6_HUMAN	2	610.4166	0.0019	0	16.46	0.055	R	LPLLR	R			5318-2-IT1-135KD.2369.236	emPAI	0.17
Q14165	MLEC_HUMAN	2	610.4166	0.0019	0	16.46	0.055	K	LPILR	S			5318-2-IT1-135KD.2369.236	emPAI	0.1
Q8TD57	DYH3_HUMAN	2	806.3844	0.0078	0	4.38	7.6	K	LAAQMQK	L	2 Deamidated (NQ); Ox	0.0002520.0	5318-2-IT1-135KD.2274.2274.2 File:~5318-2-IT1-135KD.raw~, NativeID:~controllerType=0 controllerNumber=1 scan=2274~		
Q92616	GCN1_HUMAN	2	604.3367	0.0043	0	17.94	0.13	R	VAMLR	L	Oxidation (M)	0.00500.0	5318-2-IT1-135KD.1487.1487.2 File:~5318-2-IT1-135KD.raw~, NativeID:~controllerType=0 controllerNumber=1 scan=1487~		

Legends for Table S1. List of LMP2A-IT1-interacting proteins identified by mass spectrometry. The raw wiff spectral files were processed using ProteoWizard (3.0.10577, 64-bit) to convert the data into mgf format. The resulting mgf files were then searched against the protein database using the Mascot search engine (v2.3.02) for protein identification. The table lists the 92 proteins specifically identified as potential interactors of LMP2A-IT1. Column headings are defined as follows: prot_hit_num: protein hit number retrieved from the search; prot_acc: protein accession number in the database; prot_desc: protein description; prot_score: protein score; prot_mass: molecular mass of the protein; prot_matches: number of MS/MS spectra matched to the protein; prot_matches_sig: number of MS/MS spectra with scores above the significance threshold; prot_sequences: number of unique peptides matched to the protein; prot_sequences_sig: number of unique peptides with scores above the significance threshold; prot_cover: peptide sequence coverage (%); prot_pi: theoretical isoelectric point of the protein.

Table S2

Name	sequence	Description
3RACE- adaptor primer	GCTGTCAACGATACGCTACGTAACGGCATGACAGTG TTTTTTTTTTTTTTTTTT	
3RACE outer Primer	GCTGTCAACGATACGCTACGTAACGGC	
3RACE-GSP1	GACAGTGTGGCTAAGGGAGTGTGTGC	
3RACE- GSP2	agcgtttaaacttaagctt GGGAGTGTGTGCCAGTTAAGGTG	The sequences marked in red are homology arms used for cloning into vectors
3RACE inner Primer	tttaaacgggccctctaga CGCTACGTAACGGCATGACAGTG	
5RACE-RT-GSP	CGCTGCTTTTGGGTAC	
5RACE- adaptor primer	GCTGTCAACGATACGCTACGTAACGGCATGACAGTG TTTTTTTTTTTTTTTTTTMN	M/N is a degenerate bases
5RACE outer primer	GCTGTCAACGATACGCTAC	
5RACE- GSP1	TTCTGGTTCCGGTGGA	
5RACE inner primer	agcgtttaaacttaagctt CGCTACGTAACGGCATGACAGT	The sequences marked in red are homology arms used for cloning into vectors
5RACE- GSP2	tttaaacgggccctctaga TCCGGTGGAGATGATGACGAC	
IT1-RT-GSP	ACCTTCCTAC	
LMP2A-intron Forward primer	GGGTGTGAGAGAGGGATTAAAG	
LMP2A-intron Reverse primer	CCAAGCCACACCTAACTCAT	
LMP2A-IT1 Forward primer	GTGTGGCTAAGGGAGTGTGT	
LMP2A-IT1 Reverse primer	AGGCATTACCATGTCATAGGCT	
XPO5 siRNA targeting sequence	ATGTTTGTACAGCGCTATTAA	
LMP2A-IT1 siRNA#1 targeting sequence	GAGTGTGTGCCAGTTAAGGTGATTA	
LMP2A-IT1 siRNA#2 targeting sequence	GGTTAGTCATAGTAGCTTAGCTGAA	
LMP2A-IT1 RNA FISH probe	acctttctttacttctaggc	
U1 RNA FISH probe	ctcccctgccaggtaagtat	
LacZ RNA FISH probe	ccagtgaatccgtaatcatg	
LMP2A-IT1 RISH probe(used in ARPLA)	TAAGCTACTATGACTAACCTTTCTTTACTTCTAGGCATTACCATGTCATA A AAA AAA AAATAT GAC AGA ACT AGA CAC TCT T	
U1 RISH probe(used in ARPLA)	CTG GGA AAA CCA CCT TCG TGA TCA TGG TAT CTC CCC TGC CAG GTA AGT ATA AAA AAA AAATAT GAC AGA ACT AGA CAC TCT T	

Legends for Table S2. Primers, probes, and siRNA sequences used in this study. The columns list the sequence name, the corresponding target sequence of the primer, probe, or siRNA, and a brief description of each sequence, respectively.

Fig 1A

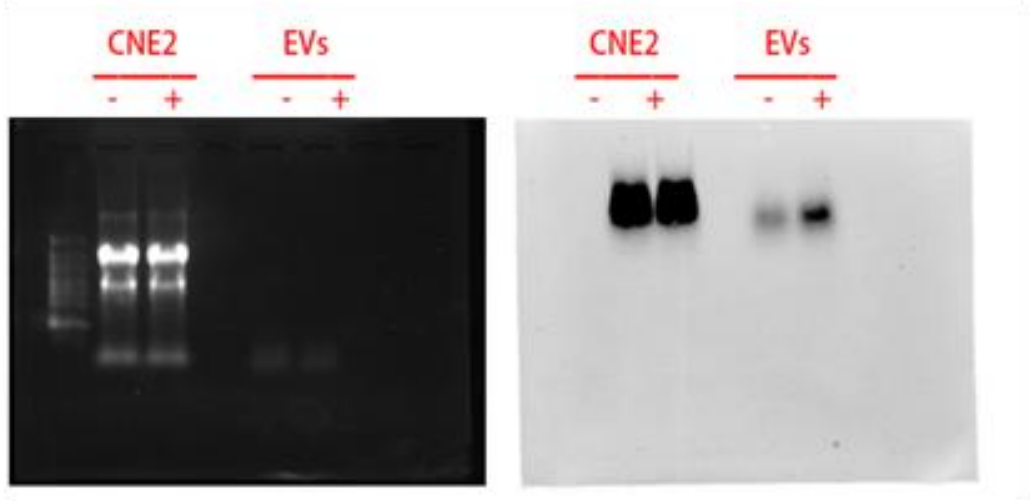

Fig 1B

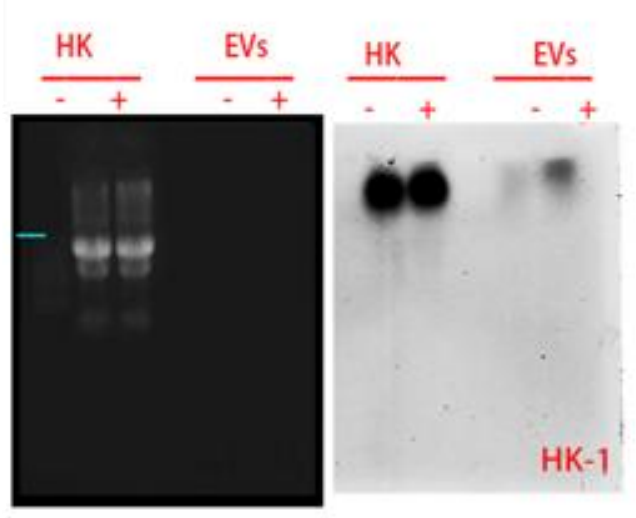

Fig 1C

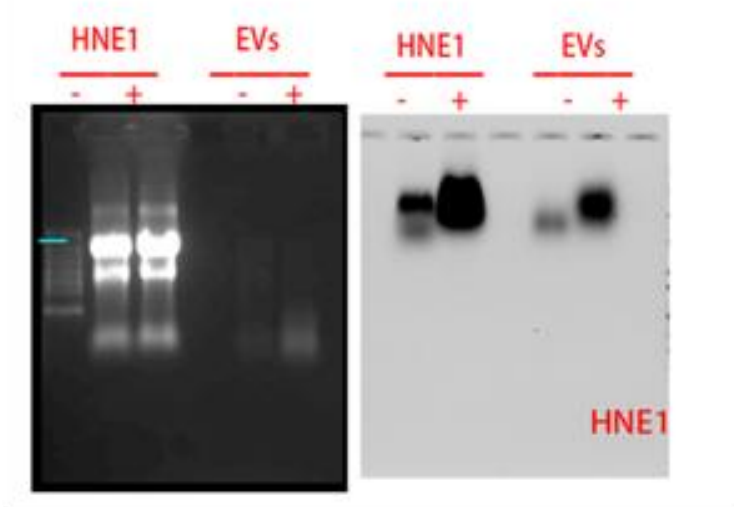

Fig 1D

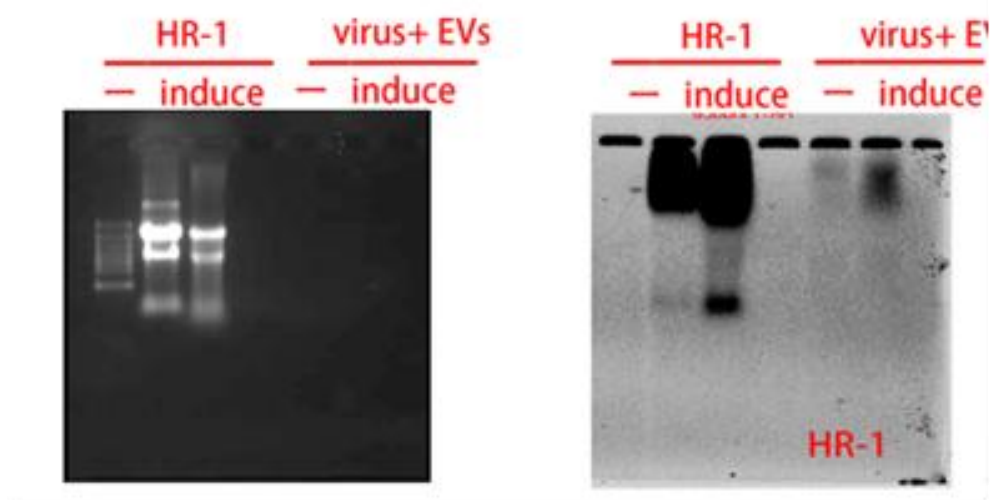

Fig 2A

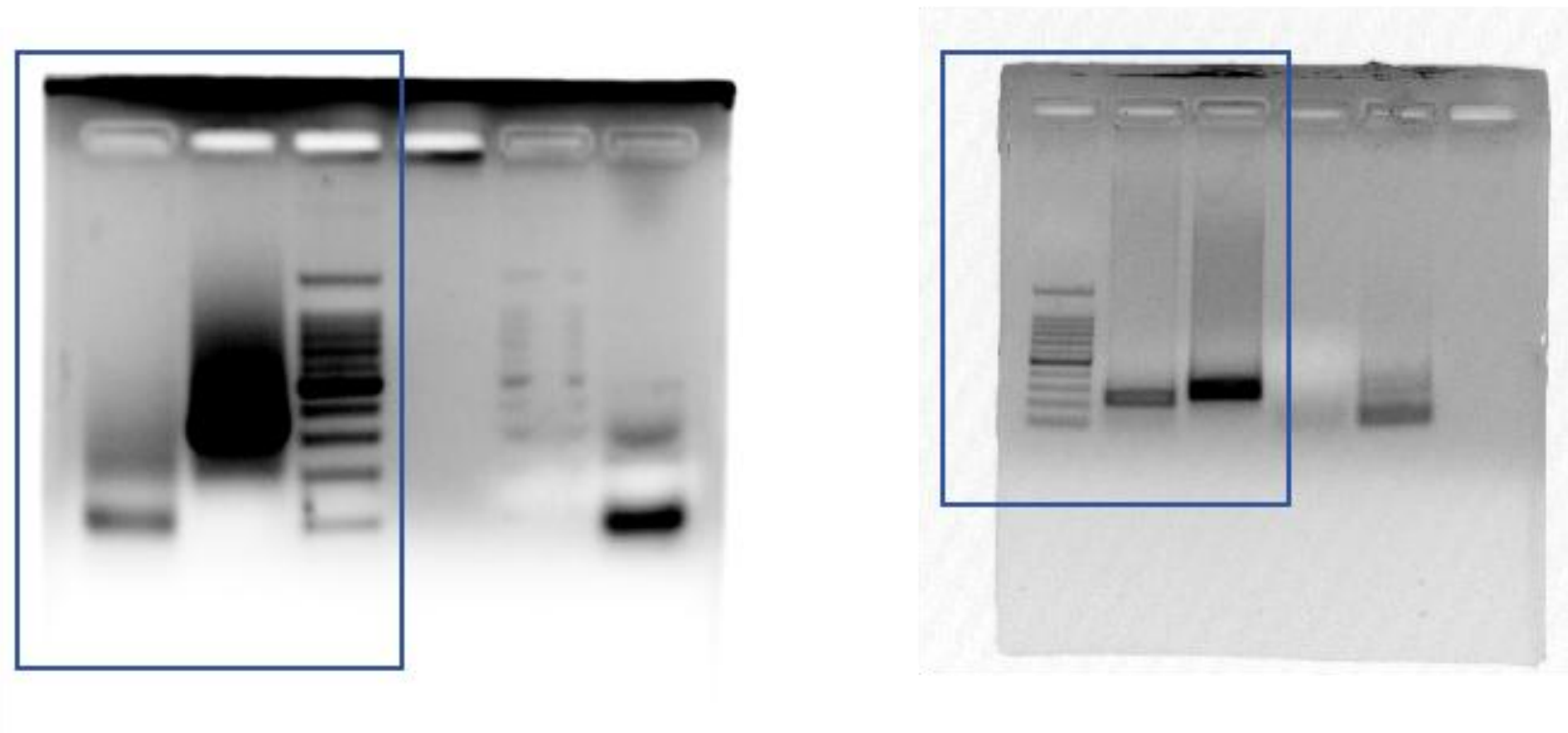

Fig 3B

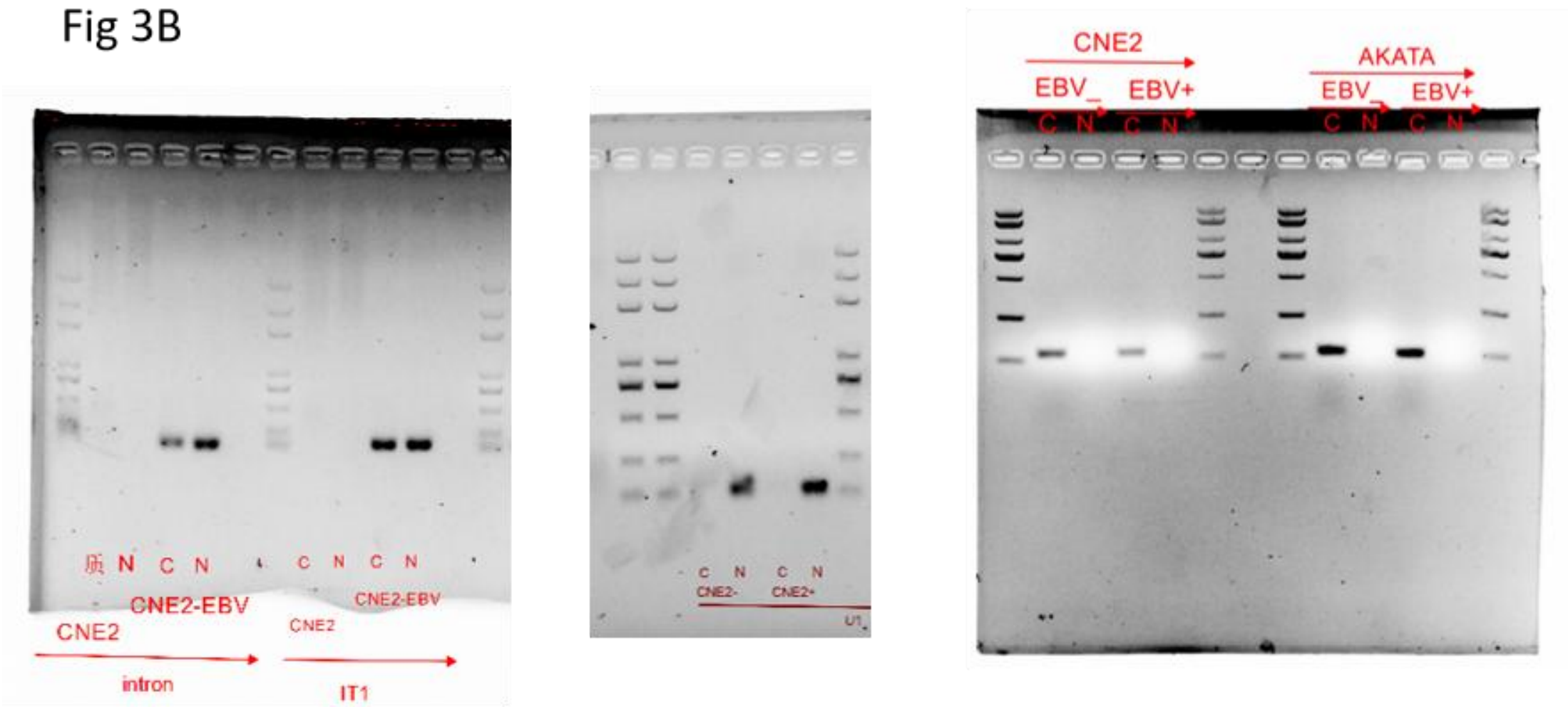

Full scans for Fig 1, 2, and 3.

Fig 3D

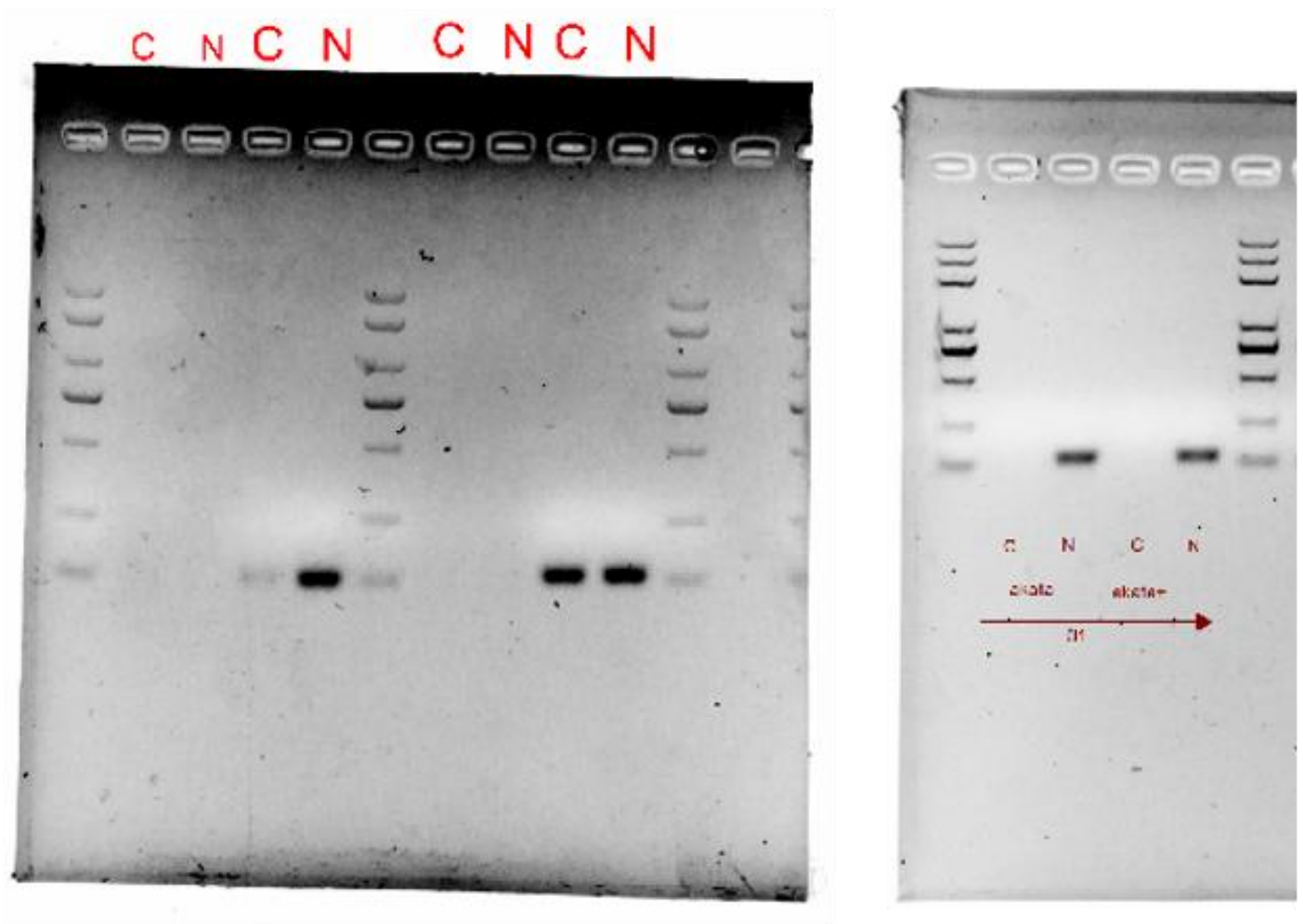

Fig 4B

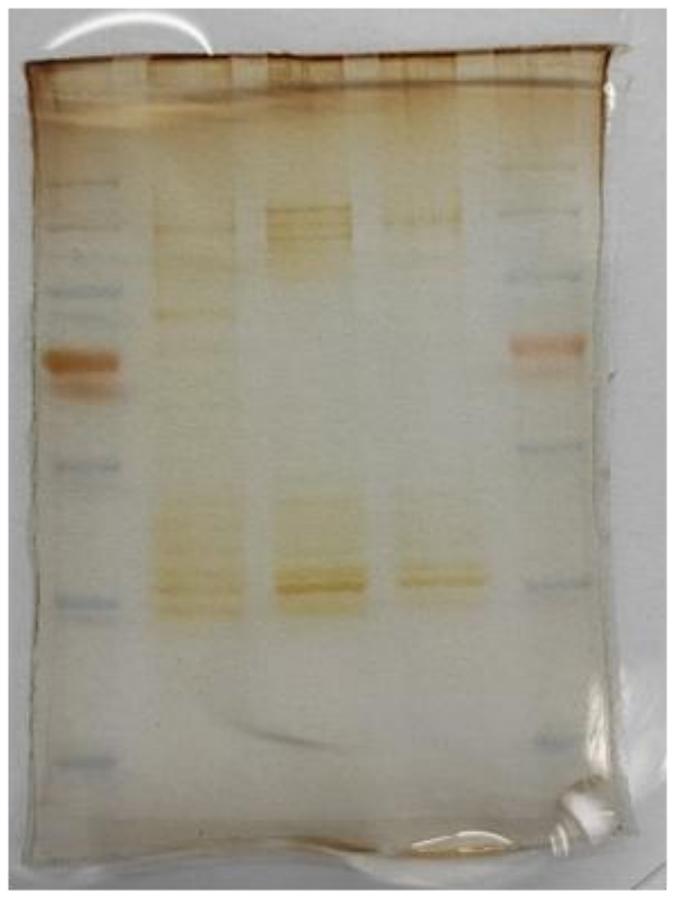

Fig 4D

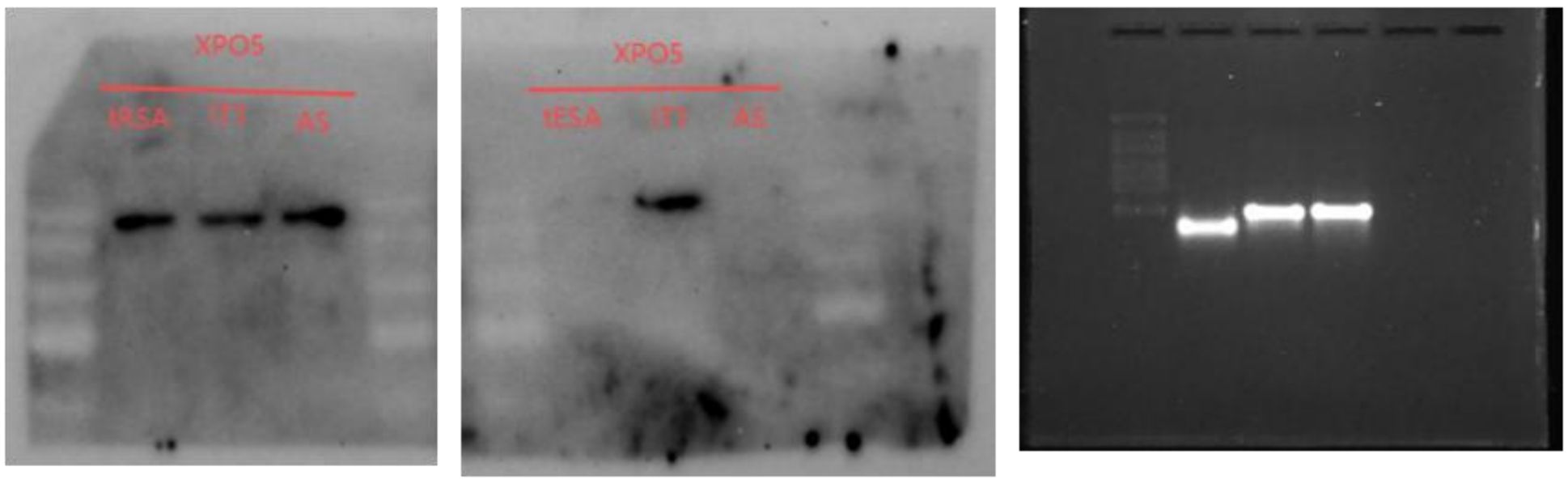

Fig 4E

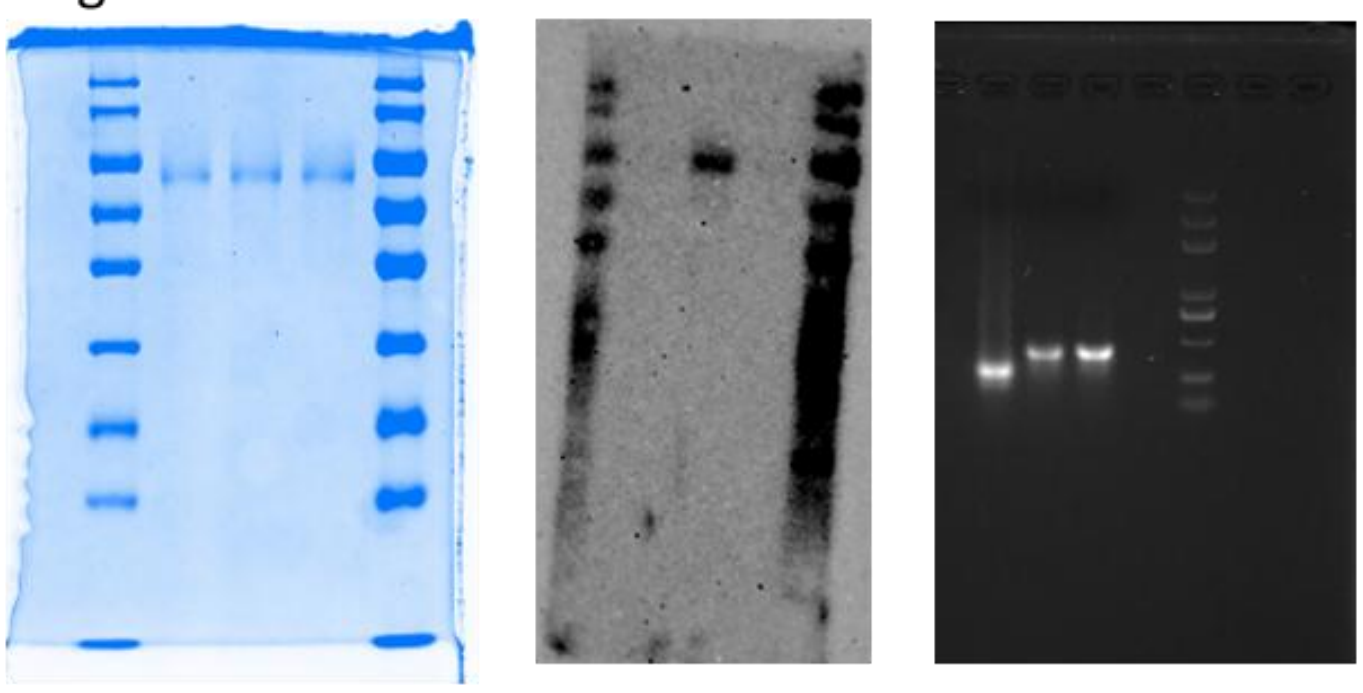

Fig 4F

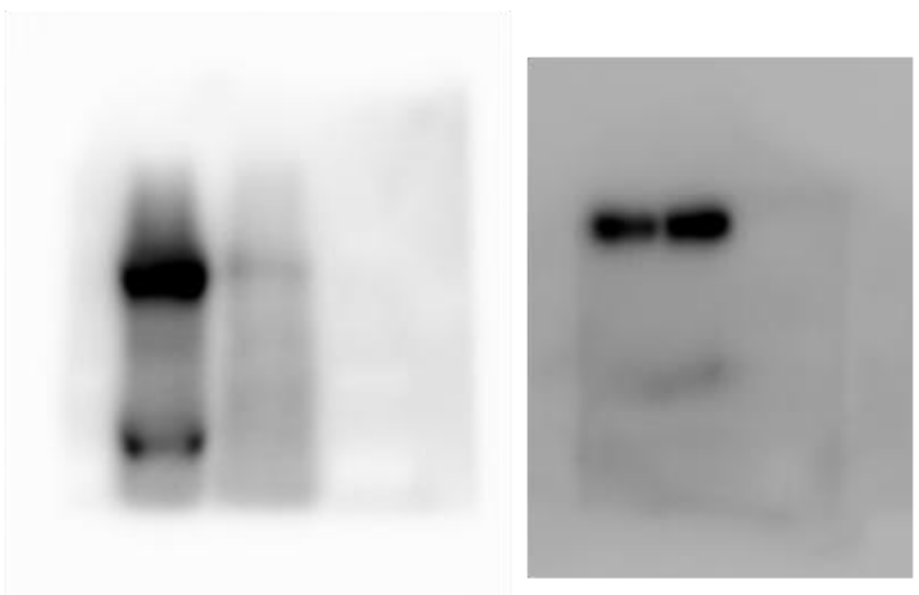

Full scans for Fig 3 and 4.

Fig 4G

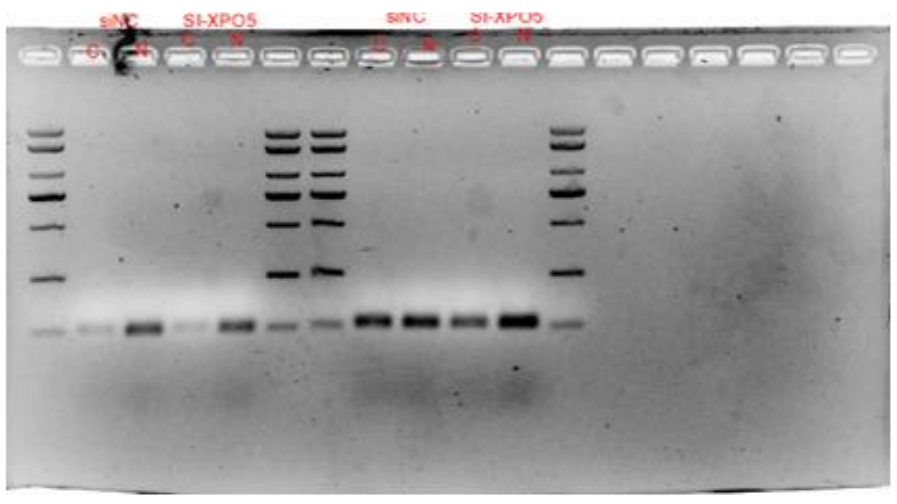

Fig 6E

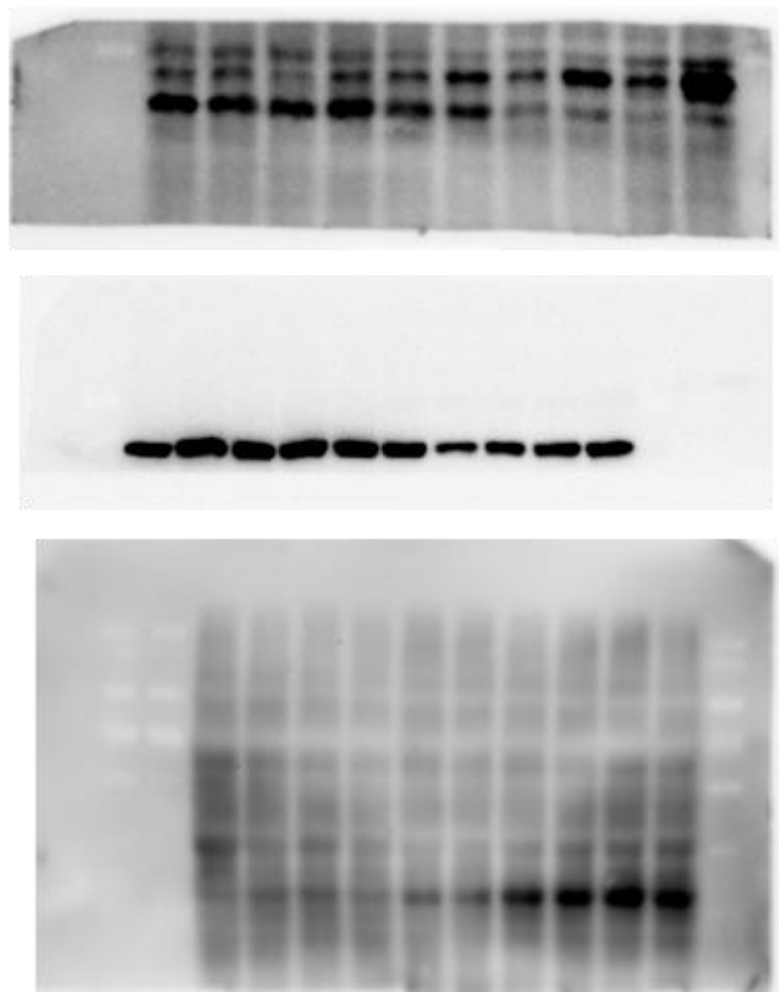

Full scans for Fig 4 and Fig 6.

The BAM files supporting the genomic visualization in Figure 1E-H are provided as independent data file named “glycoRNA BAM files.zip”.
